# Dysregulated Bone Metabolism Is Related to High Expression of miR-151a-3p in Severe Adolescent Idiopathic Scoliosis

**DOI:** 10.1155/2020/4243015

**Published:** 2020-09-26

**Authors:** Yunjia Wang, Hongqi Zhang, Guanteng Yang, Lige Xiao, Jiong Li, Chaofeng Guo

**Affiliations:** ^1^Department of Spine Surgery and Orthopaedics, Xiangya Hospital, Central South University, Changsha, China; ^2^National Clinical Research Center for Geriatric Disorders, Xiangya Hospital, Central South University, Changsha, China

## Abstract

Adolescent idiopathic scoliosis (AIS) is a common complex disease, and bone homeostasis plays an important role in its pathogenesis. Recent advances in epigenetic research show that dysregulated miRNAs may participate in the development of orthopedic diseases and AIS. The aim of this study was to detect differentially expressed miRNAs in severe AIS and elucidate the mechanism of miRNA deregulation in the pathogenesis of AIS. In the present study, miRNA expression profiles were detected in severe and mild AIS patients as well as healthy controls by miRNA sequencing. Candidate miRNAs were validated in a larger cohort. Primary osteoblasts from severe AIS patients were extracted and isolated to determine the effect of the candidate miRNAs on bone metabolism. Finally, we determined the methylation level in primary osteoblasts from severe AIS patients. The result showed that miR-151a-3p was overexpressed in severe AIS patients. Reduced GREM1 expression was observed in primary osteoblasts from severe AIS patients. miR-151a-3p directly inhibited GREM1 in primary osteoblasts. Relatively lower methylation levels were detected in primary osteoblasts from severe AIS patients. In conclusion, our study revealed that plasma miR-151a-3p levels may serve as a biomarker for severe AIS. Overexpression of miR-151a-3p may interrupt bone homeostasis via inhibiting GREM1 expression. Our result may provide a new biomarker for the early detection of AIS and increase our understanding of the pathogenesis of AIS.

## 1. Introduction

Adolescent idiopathic scoliosis (AIS) is a common complex disease affecting 0.4-5.0% children worldwide [1, 2]. The spinal curve continues to deteriorate in approximately 10% AIS patients [3]. Severe AIS patients suffer from physical and mental issues, such as severe back pain and lower self-esteem [4]. Currently, the only treatment for severe AIS is surgery, which is costly and associated with long-term implications [5]. Therefore, it is important to perform early screening and preventive intervention for early-stage AIS patients. Previous studies suggested that several factors, such as age, Risser sign, and premenarche status, serve as predictive factors for curve progression [6, 7]. However, the actual predictive power of these factors is insignificant. The reason is partly attributed to our limited understanding of the pathogenesis of AIS. Recent studies suggested that genetic factors play an important role in the pathogenesis of AIS [8]. It is important to identify several genetic and epigenetic signatures of curve progression.

miRNA is a class of small noncoding RNAs (21-25 nt) that are essential for numerous key cellular processes, such as cell differentiation and proliferation [9]. It is widely reported that miRNAs are deregulated in cancers as well as metabolic and degenerative diseases [10–12]. Recent advances in epigenetic research of AIS reveal noncoding RNAs (ncRNAs), including long ncRNAs (lncRNAs) and microRNAs (miRNAs), as promising biomarkers for early screening and prognostic predictions of AIS [13]. For instance, a recent study reported that a lowly expressed miRNA MIR4300 is related to severe AIS [14]. In another study, severely deregulated miRNAs were identified and suggested for use as biomarkers for AIS diagnosis [15]. In addition, some studies explored the mechanism underlying differentially expressed miRNAs between AIS and healthy control. A downregulated lncRNA in mesenchymal stem cells (MSCs) was associated with the suppression of osteogenic differentiation and may be involved in the pathogenesis of AIS [16]. Impaired osteocyte function in AIS was correlated with deregulated miR-145-5p/*β*-catenin signaling [17]. However, the detailed mechanism between miRNAs and severe scoliosis requires further investigation.

In the present study, miRNA expression profiles were detected in severe and mild AIS patients and healthy controls by miRNA sequencing. After bioinformatics analysis, candidate miRNAs were validated in a larger cohort. Primary osteoblasts from severe AIS patients were extracted and isolated to determine the effects of candidate miRNAs on bone metabolism. Finally, we determined the methylation level in primary osteoblasts from severe AIS patients. The aim of this study was to detect differentially expressed miRNAs in severe AIS and elucidate the mechanism of miRNA deregulation in the pathogenesis of AIS.

## 2. Materials and Methods

### 2.1. Ethics Statements

The Ethics Committee of Xiangya Hospital of Central South University approved the protocol of this study. All participants involved in this project signed a written consent form, and guardians provided a signature for subjects under the age 18 of years. All data are used for scientific research.

### 2.2. Subjects

For the miRNA sequencing cohort, all serum samples were obtained from 10 AIS patients, including five severe and five mild patients, and five healthy controls. For the qPCR validation cohort, a total 40 AIS patients and 40 healthy controls were recruited. In addition, facet joints and bone tissues of posterior column vertebrae were harvested from 21 severe AIS patients with a Cobb angle greater than 50 degree and 20 nonscoliosis patients with thoracolumbar spinal injuries during surgery. The clinical characteristics of each cohort are presented Table 1. Two spinal surgeons confirmed the diagnosis of each AIS patient according to physical examination and the standard anteroposterior radiography method. In this study, severe AIS patients exhibited a maximum Cobb angle of greater than 50°, and mild AIS patients exhibited a maximum Cobb angle less than 40°. All patients with familial inherited diseases, congenital vertebral malformations, neuromuscular diseases, skeletal dysplasia, and connective tissue abnormalities were excluded from the study.

### 2.3. RNA Extraction and Small RNA Sequencing

A peripheral venous blood sample was aspirated from each subject in the morning, and the plasma was isolated and stored in an -80°C freezer for future analysis. Total cell-free RNA was extracted from plasma using the miRNeasy Serum/Plasma kit (Qiagen, CA, USA) per the protocol's instructions. A small RNA library was established by using TruSeq the Small RNA Sample Prep Kit (Illumina, San Diego, CA, USA). Then, the qualified library was loaded on an Illumina HiSeq 2500 platform for high-throughput sequencing (Lianchuan Biotechnology, Hangzhou, China). The raw data of each sample were filtered by ACGT101-miR (LC Sciences, Houston, Texas, USA) to remove junk sequences. Next, all of the 18- to 26-nucleotide sequences were mapped to the miRBase21.0 database (http://www.mirbase.org/) to identify known miRNAs and novel 5p or 3p miRNAs [18]. Differentially expressed miRNAs were assessed using an analysis of variance (ANOVA) test. To predict the target genes of each miRNAs, TargetScan7.2 (http://www.targetscan.org/vert_72/) and miRDB (http://mirdb.org/) databases were used to identify the potential binding site [19, 20].

### 2.4. Real-Time Quantitative PCR (RT-PCR)

Total RNA was extracted from the bone tissue of the facet joint, posterior column vertebrae, or primary osteoblasts using TRIzol reagent (Invitrogen, Thermo Fisher Scientific) according to the protocol's instruction. A miRNA cDNA Synthesis Kit (Thermo Fisher Scientific) was used for miRNA reverse transcription. In brief, an adaptor sequence, i.e., 3′ poly-A tailing and 5′ ligation, was added to mature miRNA. Then, a standard reverse transcription reaction was performed. For mRNA reverse transcription, cDNA was synthesized using the TaqMan cDNA synthesis kit (Applied Biosystems, CA, USA) following the manufacturer's protocol. All primers used in this study are provided in supplemental table S2. Real-time PCR was conducted by using the SYBR Green Master Mix (Takara, Kusatsu, Shiga, Japan) according to the manufacturer's protocol. GAPDH and U6 were used as internal controls.

### 2.5. Isolation and Culture of Primary Osteoblasts

For human primary osteoblast isolation, chopped cancellous bone tissues from the facet joint or posterior column vertebrae were isolated as described before [21]. In brief, samples were washed with phosphate-buffered solution (PBS) thrice and then incubated with 0.25% trypsin (Gibco, CA, USA) for 30 min at 37°C. Samples were centrifuged after complete medium was added. The supernatant was discarded, and the pellet was resuspended in 0.1% collagenase type I (Sigma-Aldrich, St. Louis, MO, USA). The sample was incubated in a 37°C incubator for 4 h and then cultured with F-12 medium (Gibco, Carlsbad, CA, USA) supplemented with 10% fetal bovine serum (FBS) in an incubator (37°C and 5% CO_2_).

### 2.6. Western Blot

Total cellular proteins from primary osteoblasts were collected in lysis buffer with protease inhibitors. Protein samples were then sonicated and boiled for 5 min. After quantification using the BCA protein assay (Thermo Fisher Scientific, MA, USA), twenty micrograms of each protein sample were loaded into a 10% SDS gel for separation and then transferred to a PVDF membrane (Bio-Rad, CA, USA). The membranes were blocked with skim milk for 1 h at room temperature and incubated with antibodies against GREM1 (1 : 2000, HPA007526, Sigma-Aldrich) overnight at 4°C on a shaker. After three washes in PBST, the membrane was incubated with goat anti-rabbit IgG antibody (1 : 10000, ab150077, Abcam) for 1 h at room temperature. The blot was detected using the Chemiluminescence Protein Detection Module (Millipore, CA, USA). GAPDH (1 : 5000, EPR16891, Abcam) was used as an internal control. Band intensity was measured using the ImageJ (v 1.52p; National Institutes of Health) program.

### 2.7. Luciferase Reporter Assay

TargetScan7.2 and miRDB databases were used to identify the binding site between miR-151a-3p and GREM1. GREM1 3′UTR and 5-base mutated GREM1 3′UTR regions were cloned into the luciferase reporter plasmid (pGL4.26, Promega Corporation, WI, USA). Reporter plasmid and miR-151a-3p mimic or negative control mimics were cotransfected into HEK293-T cells using Lipofectamine 2000. After 24 h incubation, luciferase activities were determined using the Dual-Luciferase Reporter assay system (Promega, WI, USA) according to the manufacturer's protocol.

### 2.8. Cell Transfection

Primary osteoblasts were transfected with miR-151a-3p mimic, miR-151a-3p inhibitor, or negative controls using Lipofectamine 2000. In brief, cells were plated in a six-well plate at a density of 1 × 10^5^ cells/well 24 h before transfection. Then, cells were transfected with 50 nM of miR-151a-3p mimic, inhibitor, or negative control in a 37°C and 5% CO_2_ incubator for 4 h. The sequences used for transfection were as follows: miR-151a-3p mimic, 5′-GCTAAACTAACCCTCCTGTCAGCCC-3′; miR-151a-3p inhibitor, 5′-CUAGACUGAAGCUCCUUGAGG-3′; and negative control, 5′-UUCUCCGAACGUGUCACGUTT-3′. In the transfection experiments, primary osteoblasts were incubated with 50 ng/ml BMP-2 (H4971, Sigma-Aldrich, MO, USA). After 48 h incubation, primary osteoblasts were harvested for qPCR assessment of GREM1 mRNA.

### 2.9. Staining

Alizarin Red staining was conducted after primary osteoblasts were transfected with miR-151a-3p mimic and incubated with 50 ng/ml BMP-2 for two weeks. Briefly, cells were cultured in six-well plates, incubated with BMP-2 and transfected with miR-151a-3p mimic. After two weeks, the cells were fixed with 4% paraformaldehyde (PFA) for 30 min. After three washes in PBS, cells were incubated with 0.4% Alizarin Red S (Sigma-Aldrich, MO, USA) for 10 min. Staining was assessed by microscopy. The calcium concentration was determined using 10% cetylpyridinium chloride (CPC) (Sigma-Aldrich, MO, USA). Cells were incubated with 10% CPC for 15 min at room temperature. Then, the diluted sample was transferred into a 96-well plate. Absorbance at 562 nm was detected using a plate reader (BD Biosciences, CA, USA).

### 2.10. Methylation-Specific PCR (MSP)

Bisulfite conversion of genomic DNA was performed using the EpiTect Fast Bisulfite Conversion Kit (Qiagen) per the protocol's instruction. Modified DNA was used as a template for MSP (primer sequences for MSP listed in supplemental table S5). The following PCR protocol for methylated and unmethylated miR-151a was employed: preheating at 94°C for 5 min; 40 cycles of 94°C for 30 s, annealing at 56°C for 30 s, and extension at 72°C for 45 s; and 72°C for 10 min. PCR products were separated by 1% agarose gel electrophoresis. The methylation status of each sample is determined based on the presence of a visible band with the methylated primer (M) regardless of the presence of a band with the unmethylated primer (U). However, the unmethylated status is determined based on the presence of the U band and the absence of the M band.

### 2.11. Statistical Analysis

Results are presented as the mean values ± standard deviation (SD) and analyzed using MedCalc (MedCalc Software, v16.0, Belgium). The Mann–Whitney *U* test was performed to compare the anthropometric data from the AIS and control groups. Receiver operating characteristic (ROC) curve analysis was conducted to determine the efficiency of each miRNA in predicting progression of the Cobb angle. Two-sided Student's *t*-tests were performed to compare experimental results from the AIS and control groups. *P* values < 0.05 were considered statistically significant.

## 3. Result

### 3.1. Differentially Expressed MicroRNA Profiles

**Figure 1:**
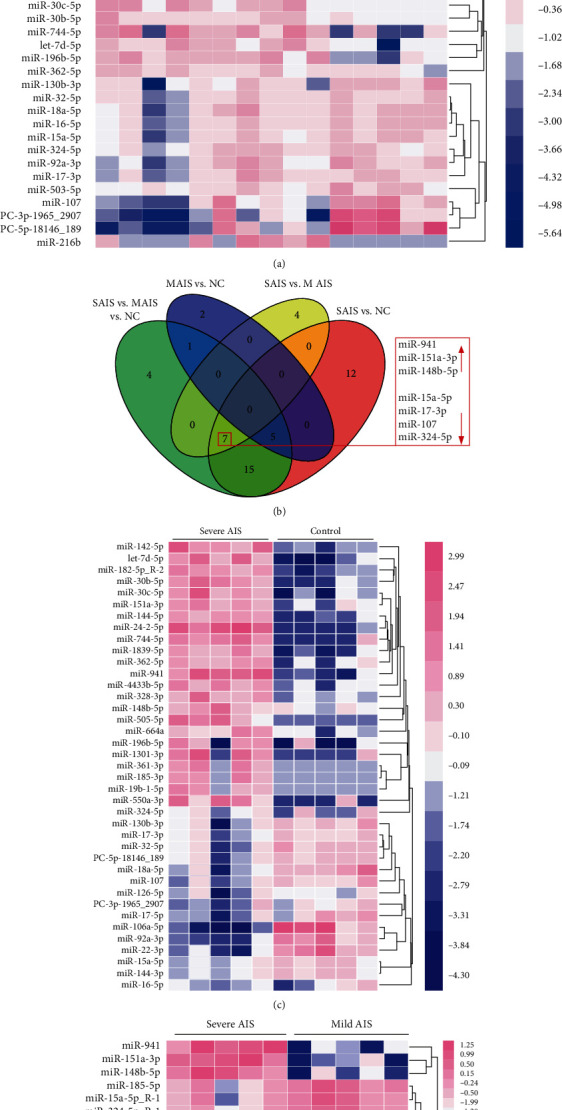
Differentially expressed miRNAs in plasma from severe, mild scoliosis and healthy controls. (a) miRNA sequencing was performed in severe AIS, mild AIS, and healthy control groups, and each group has five samples. In total, 32 significantly differentially expressed miRNAs were represented in the heat map. Hierarchical clustering analysis revealed that two major clusters exist between the three groups. Pink and blue represent upregulated and downregulated expressions, respectively. (b) Venn diagram revealed the convergence of differentially expressed miRNAs between comparisons. S AIS represents the severe AIS group, M AIS represents the mild AIS group, and NC represents the healthy control group. (c) Heat map of differentially expressed miRNAs between the severe AIS and healthy control groups. Pink and blue represent upregulated and downregulated expressions, respectively. (d) Heat map of differentially expressed miRNAs between the severe AIS and mild AIS groups. Pink and blue represent upregulated and downregulated expressions, respectively.

To identify differentially expressed microRNAs among severe and mild AIS patients and nonscoliosis controls, we performed plasma miRNA deep sequencing on a cohort of five severe AIS patients, five mild AIS patients, and five healthy controls. The clinical characteristics of three groups were similar with the exception of the main curve Cobb angle (Table 1). The results indicated that 32 miRNAs were differentially expressed in the three groups (*P* < 0.05) (Figure 1(a)). In total, 39 miRNAs were differentially expressed in the severe AIS group compared with the healthy control group, including 23 upregulated and 16 downregulated miRNAs (Figure 1(c), supplemental table S1). Comparing the severe AIS group with the mild AIS group, 11 miRNAs were differentially expressed, including three upregulated and eight downregulated miRNAs (Figure 1(d)). Eight miRNAs were differentially expressed, including seven upregulated and one downregulated miRNAs, in the mild AIS group compared with the healthy control group. Venn diagram analysis revealed that no shared differentially expressed miRNAs were noted between the mild AIS vs. control groups and severe vs. mild AIS groups, indicating that no representative miRNAs for the mild AIS group can be detect in our cohort (Figure 1(b)). Regarding the severe AIS group, seven miRNAs were shared by all the groups with the exception of mild AIS vs. the control group. Among all the seven miRNAs, three were upregulated in each profile: miR-941, miR-151a-3p, and miR-148b-5p.

### 3.2. Validation of Candidate miRNAs in a Larger Cohort

**Figure 2:**
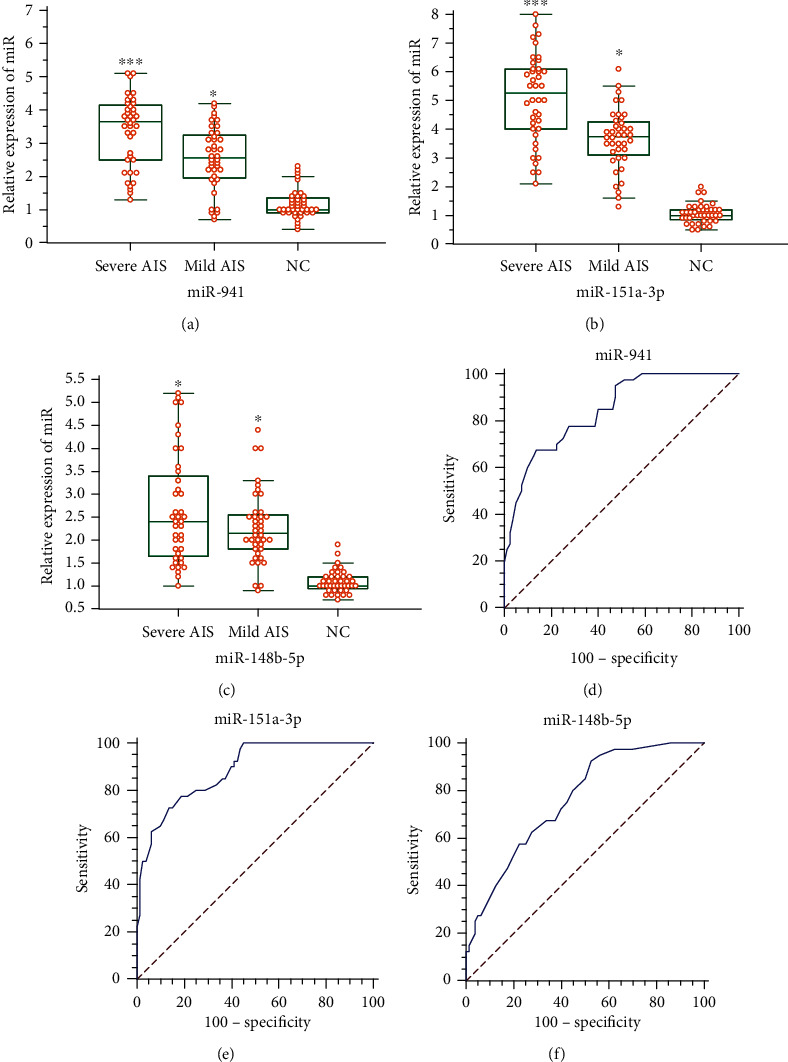
Expression levels of candidate miRNAs in the verification cohort. (a–c) Relative expression levels of miR-941, miR-151a-3p, and miR-148b-5p from plasma samples of the validation cohort, which consist of 40 samples from each group were determined by quantitative real-time PCR. The mean value of the normal control group was set to 1.0. Data are displayed as the mean ± SD. ^∗^*P* < 0.05 versus the NC group and ^∗∗^*P* < 0.05 versus the mild AIS group. NC = normal control. (d–f) The receiver operator characteristic (ROC) curve for plasma miR-941, miR-151a-3p, and miR-148b-5p levels to predict greater Cobb angle was analyzed. The area under the curve (AUC) was 0.845, 0.885, and 0.762 (corresponding to 95% confidence intervals are 0.767-0.904, 0.815-0.936, and 0.676-0.835), respectively.

To identify the expression pattern of candidate miRNAs among severe, mild AIS patients, and healthy controls, quantitative real-time PCR was performed to determine plasma expression levels of miR-941, miR-151a-3p, and miR-148b-5p in a larger verification cohort consisting of 40 severe AIS patients, 40 mild AIS patients, and 40 healthy controls. The clinical characteristics of the validation cohort are presented in Table 1. The results demonstrated that miR-941 and miR-151a-3p expression levels are significantly altered in the three groups (Figures 2(a) and 2(b)). Significantly increased expression of these two miRNAs is noted in the mild AIS group compared with the control group. However, the miR-148b-5p expression pattern was not consistent with miRNA sequence results (Figure 2(c)). The predictive power of these miRNAs for progressive scoliosis was calculated based on receiver operating characteristic (ROC) analysis. The area under the curve (AUC) values for each miRNA were 0.845, 0.885, and 0.762 (corresponding 95% confidence intervals are 0.767-0.904, 0.815-0.936, and 0.676-0.835) (Figures 2(d)–2(f)). In addition, miR-151a-3p exhibits the highest expression differences among the three groups and the most powerful diagnostic accuracy for severe AIS among all these three miRNAs. Thus, miR-151a-3p was selected as the candidate miRNA involved in the development of progressive AIS and used in subsequent studies.

### 3.3. Target Prediction of miR-151a-3p and Expression Level Validation

**Figure 3:**
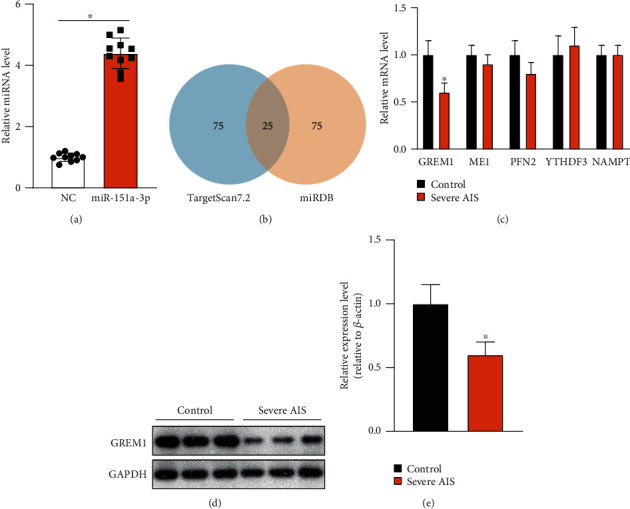
Expression levels of miR-151a-3p and GREM1, a predicted target gene of miR-151a-3p, in spinal bone tissues from severe scoliosis patients. (a) Total RNA was extracted from the cancellous bone of the facet joint harvested from severe AIS patients undergoing corrective surgery and nonscoliosis patients undergoing spine surgery. Relative miRNA expression levels of miR-151a-3p were determined by quantitative real-time PCR. (b) Venn diagram analysis of target genes predicted by TargerScan7.2 and miRDB. (c) Relative mRNA expression levels of GREM1, ME1, PFN2, YTHDF3, and NAMPT from total RNA extracted from the cancellous bone of severe AIS and nonscoliosis patients. (d, e) GREM1 protein expression in primary osteoblasts extracted from severe AIS and nonscoliosis patients was analyzed by Western blotting. GAPDH was used as an internal reference. Gray value quantitative analysis was performed and reported. The mean value of the nonscoliosis group was set to 1.0. Data are displayed as the mean ± SD. ^∗^*P* < 0.05 versus the NC group (*n* = 3).

To further verify the relationship between miR-151a-3p and progressive AIS, we obtained facet joint and other bone structures from postcolumn vertebrae from severe AIS patients during corrective surgery and extracted the total RNA and protein from the cancellous bone of the facet joint and vertebral plate. We determined miR-151a-3p expression levels by quantitative real-time PCR. The miR-151a-3p levels of AIS patients are significantly increased compared with nonscoliosis controls (Figure 3(a)). To identify the downstream target gene of miR-151a-3p in AIS, we employed two miRNA target prediction databases, TargetScan7.2 and miRDB, to predict the potential targets of miR-151a-3p (supplemental table S3, 4). Among the top 100 predicted targets of each database, 25 genes were the same as demonstrated by the Venn diagram (Figure 3(b)). We screened these 25 genes based on gene function and expression pattern since most of them are not involved in skeletal development or expressed in bone tissue. Finally, five predicted target genes were identified as potential genes that interact with miR-151a-3p, including GREM1, ME1, PFN2, YTHDF3, and NAMPT. Then, we determined mRNA expression levels of these genes in posterior column vertebrae from severe AIS patients. In AIS patients, only GREM1 is significantly downregulated compared with the control group (Figure 3(c)). To further confirm this result, we isolated primary osteoblasts from bone tissues of the posterior column vertebrae and determined GREM1 protein expression levels in primary cells. Reduced GREM1 protein levels were noted in AIS patients (Figures 3(d) and 3(e)).

### 3.4. GREM1 Is Directly Regulated by miR-151a-3p

**Figure 4:**
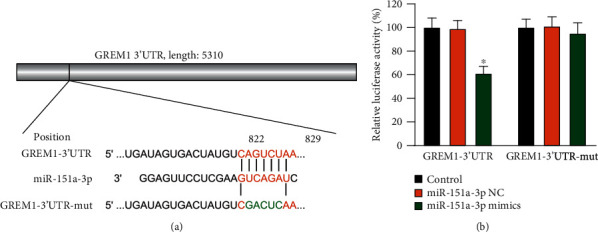
Validation of miR-151a-3p targeting GREM1 mRNA. (a) Schematic figure demonstrating alignment of miR-151a-3p, wild-type, and mutant GREM1 3′UTR sequences. (b) Luciferase reporter assay was performed after HEK-293T cells were cotransfected with a random sequence. miR-151a-3p or control mimic as well as the plasmid vector with wild-type or mutant GREM1 3′UTR. ^∗^*P* < 0.05 versus the control group (*n* = 3).

To further elucidate the interaction between miR-151a-3p and GREM1, luciferase reporter assays were performed by cotransfecting a wild-type or mutant GREM1 luciferase reporter vector with miR-151a-3p mimic into HEK-293T cells. The potential binding site and mutant GREM1 reporter vector sequence is reported (Figure 4(a)). The level of fluorescence was significantly decreased in cells cotransfected with the wild-type GREM1 3′UTR plasmid and the miR-151a-3p mimic (Figure 4(b)). The results indicate that GREM1 is directly regulated by miR-151a-3p.

### 3.5. Overexpression of miR-151a-3p Inhibits Mineralization of Primary Osteoblasts by Suppressing GREM1 Expression

**Figure 5:**
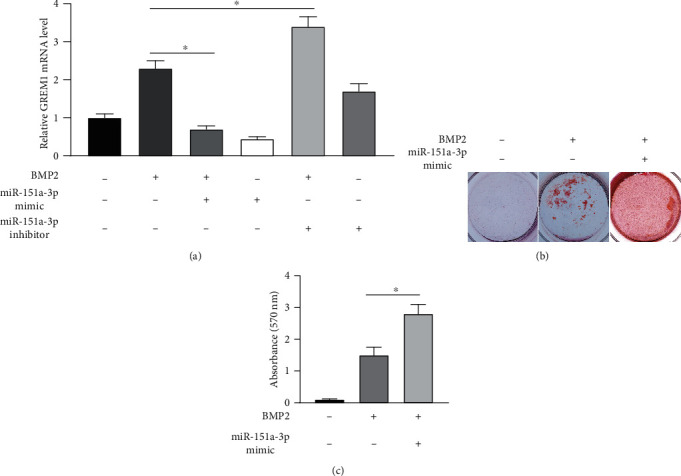
GREM1 expression was inhibited by miR-151a-3p in primary osteoblasts. (a) Primary osteoblasts extracted from severe AIS samples were incubated with or without BMP2 and transfected with miR-151a-3p mimic or inhibitor. GREM1 mRNA expression levels were determined by quantitative real-time PCR. The mean value of the untreated group was set to 1.0. ^∗^*P* < 0.05 between the indicated two groups (*n* = 3). (b) Mineralization of primary osteoblasts incubated with BMP2 and transfected with miR-151a-3p mimic for two weeks was detected and quantified by Alizarin Red S staining. (c) Quantification of mineralization was performed. ^∗^*P* < 0.05 between the indicated two groups (*n* = 3).

GREM1 expression can be stimulated by BMP2. Thus, GREM1 exhibits a BMP2 antagonist effect. The balance between these two factors is necessary for skeletal homeostasis. To elucidate the detailed role of elevated miR-151a-3p expression in AIS progression, we first evaluated the effect of miR-151a-3p on GREM1 mRNA expression in primary osteoblast incubated with 50 ng/ml BMP2. BMP2 incubation increased GREM1 mRNA expression levels, which is consistent with previous studies. Overexpression or inhibition of miR-151a-3p along with BMP2 incubation significantly decreased or increased GREM1 expression (Figure 5(a)). Then, we performed Alizarin Red staining to evaluate the mineralization level of primary osteoblasts incubated with 50 ng/ml BMP2 with or without 50 nM miR-151a-3p mimic transfection for two weeks. The results showed that miR-151a-3p overexpression combined with BMP2 incubation significantly increased the mineralization of AIS primary osteoblasts (Figures 5(b) and 5(c)).

### 3.6. Promoter Methylation Status of miR-151a-3p in Primary Osteoblasts from Severe AIS Patients

**Figure 6:**
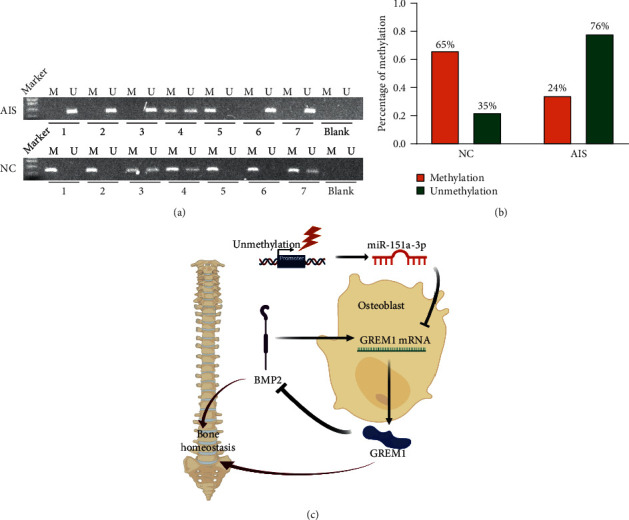
Methylation status of miR-151a in primary osteoblasts from severe AIS and controls. (a) Methylation status of miR-151a in primary osteoblasts from severe AIS patients and controls was determined by methylation-specific PCR. M: methylated; U: unmethylated. (b) Methylation percentage in samples was calculated and reported. (c) Graphic illustration of this study.

High miRNA expression is caused by a variety of mechanisms, such as unmethylated CpG islands the in promoter region. Here, we assessed the promoter methylation status of miR-151a by methylation-specific PCR (MSP) in primary osteoblasts from 21 progressive AIS patients and 20 nonscoliosis patients. We found that 65% (*n* = 13 out of 20) of nonscoliosis samples exhibited miR-151a promoter methylation. However, only 24% (*n* = 5 out of 21) of AIS samples exhibited promoter methylation, and this level is reduced compared with the nonscoliosis control group (Figures 6(a) and 6(b)).

## 4. Discussion

AIS is a complex disease, and genetic factors play an important role in its pathogenesis. Although previous studies have reported many causative genes and related underlying mechanisms [22], it is still difficult to predict scoliosis development and progression in adolescents. In the current study, we performed microRNA sequence analysis in AIS patients with severe and mild spine curvature, which is typically referred to as the Cobb angle and identified miRNAs associated with progressive AIS. We next identified miR-151a-3p as a biomarker for severe AIS using bioinformatics analysis combined with expression validation. Here, miR-151a-3p may also contribute to the development of a greater Cobb angle via inhibition of GREM1 expression in osteoblasts to interrupt bone homeostasis, which is an influential factor in spine development. Finally, we observed relatively lower methylation levels in primary osteoblasts from severe AIS patients, which may cause high miR-151a-3p expression levels. This study linked the imbalance in epigenetic regulation with severe AIS, which might partially explain why mutations have not been identified in a large proportion of AIS patients.

The relationship between miRNA and AIS has been described in a few recent papers. In a study based on RNA sequencing data from plasma samples, circulating miR-122-5p, miR-27a-5p, and miR-223-5p levels were upregulated in AIS patients [15]. In addition, another study identified seven highly expressed miRNAs in bone marrow mesenchymal stem cells (BM-MSCs) from AIS patients based on a microarray approach [23]. All these results suggested that miRNAs participate in the pathogenesis of AIS. We profiled miRNA differences among severe AIS, mild AIS, and normal controls. The results indicated that no specific miRNA could serve as a biomarker for mild AIS, but seven miRNAs significantly correlated with severe AIS. It is possible that deregulated miRNAs are exclusively involved in the progression of imbalanced spinal growth; this hypothesis needs further confirmation. Therefore, only severe AIS patients with a Cobb angle greater than 50 degrees were recruited for validation and mechanistic experiments. However, only a few of the candidate miRNAs have been functionally verified. In a study of primary osteoblasts from AIS patients, miR-145 overexpression in the bone tissue was revealed by microarray analysis. Knockdown experiments showed that miR-145 might contribute to bone loss in AIS by targeting osteocytes [17]. Statistics showed that most of the verified miRNAs are pertinent to processes involved in musculoskeletal system development, such as osteogenic differentiation of BM-MSCs and osteoblastic function [13]. In the present study, we introduced two miRNA target prediction methods to localize the specific target of miR-151a-3p in primary osteoblasts from severe AIS patients. Many potential genes still need to be confirmed. Therefore, we screened target genes based on gene function and expression. Genes associated with bone metabolism or expressed in musculoskeletal tissue were retained for subsequent qPCR validation.

Gremlin-1 (GREM1), a differential screening-selected (DAN) family protein, is a bone morphogenetic protein (BMP) antagonist that directly binds to BMPs, including BMP-2, BMP-4, and BMP-7, to regulate skeletal patterning and homeostasis [24, 25]. BMP-2 promotes mesenchymal stem cell (MSC) differentiation into bone-related cells during embryogenesis and development and maintains bone tissue homeostasis in adults [26]. More importantly, GREM1 expression in osteoblasts is induced by BMPs [27]. Given that GREM1 expression decreased in primary osteoblasts from AIS patients, we incubated BMP-2 with miR-151a-3p mimic transfection to determine the interaction between miR-151a-3p and GREM1. In previous studies, *Grem1* overexpression inhibits osteoblastogenesis and causes osteopenia in mice [28]. However, osteopenia, particularly at the vertebral region, and skeletal abnormalities were demonstrated in *Grem1* null mice in which *Grem1* was globally inactivated. This phenomenon is also observed with other BMP antagonists, such as *connective tissue growth factor* (Ctgf) *[*29*]*. These manifestations suggest that excessive levels of BMP antagonists suppress the effect of BMP on bone development, but low levels of BMP antagonists are necessary for bone homeostasis maintenance. In the current study, mineralization of primary osteoblasts was enhanced after miR-151a-3p mimic transfection. However, the clinical manifestations of enhanced osteogenesis, such as high bone density, were not found in our severe AIS cohort. Consequently, the increased miR-151a-3p levels observed in severe AIS patients may impair bone homeostasis by inhibiting GREM1; however, comprehensive alteration of bone metabolism must be further investigated.

Several limitations in our study should be noted. First, the observations made in plasma samples and primary osteoblasts likely do not comprehensively represent the scenario in patients. miR-151a-3p or GREM1 may impact other aspects of skeletal metabolism, which require other tissues and larger samples for validation. Second, we extracted primary osteoblasts to investigate the effect of miR-151a-3p. However, it has been widely reported that BMP-2 and GREM1 exhibit regulatory effects on MSCs. Thus, it is necessary to explore the effect of miR-151a-3p on MSCs. Finally, the relationship between bone homeostasis and the development and progression of scoliosis remains largely unknown. We were unable to elucidate the detailed mechanism underlying the effect of GREM1 on spinal development.

In conclusion, our study revealed that plasma miR-151a-3p might serve as a biomarker for severe AIS. The overexpression of miR-151a-3p may contribute to the progression of scoliosis via inhibition of GREM1 expression in osteoblasts to interrupt bone homeostasis. Finally, relatively lower methylation levels might explain high miR-151a-3p levels (Figure 6(c)). Our result may provide a new biomarker for early detection of AIS, and these results deepen our understanding of the pathogenesis of AIS.

## Figures and Tables

**Table 1:** Physical characteristics of the AIS groups and control group.

Cohort I (miRNA sequence)			
Items	Severe AIS (*n* = 5)	Mild AIS (*n* = 5)	Control (*n* = 5)
Age (yr)	13.0 ± 2.5	14.2 ± 0.8	11.6 ± 2.9
Gender (female/male)	5/0	5/0	5/0
Weight (kg)	42.4 ± 7.0	45.0 ± 2.6	39.5 ± 10.1
Height (cm)	152.4 ± 8.8	157.8 ± 3.6	148.6 ± 15.2
BMI (kg/m^2^)	18.1 ± 1.3	18.1 ± 0.4	17.6 ± 0.9
Cogg angle (°)	64.6 ± 16.7‡	26.0 ± 5.8	—
Cohort II (qPCR validation)			
	Severe AIS (*n* = 40)	Mild AIS (*n* = 40)	Control (*n* = 40)
Age (yr)	12.6 ± 2.2	12.4 ± 1.8	12.2 ± 1.9
Gender (female/male)	27/13	30/10	28/12
Weight (kg)	42.1 ± 7.3	39.6 ± 5.3	39.6 ± 6.6
Height (cm)	153.1 ± 10.4	150.2 ± 7.8	150.8 ± 9.1
BMI (kg/m^2^)	17.8 ± 1.2	17.5 ± 1.4	17.3 ± 1.4
Cogg angle (°)	61.3 ± 9.5‡	22.7 ± 6.7	—

Values are shown as the means ± SD. AIS: adolescent idiopathic scoliosis; BMI: body mass index. ^†^*P* < 0.05 and ^‡^*P* < 0.01 between the severe AIS group and mild AIS group; ^∗^*P* < 0.05 and ^∗∗^*P* < 0.01 between the severe AIS group and control group.

## Data Availability

The datasets used and/or analyzed during the current study are available from the corresponding author on reasonable request.

## Supplementary Materials

Supplementary Materials Supplemental table S1: candidate miRNA expression profile by miRNA sequencing. Supplemental table S2: summary of the primer sequences for RT-PCR. Supplemental table S3: predicted target gene of miR-151a-30 by TargetScan7.2. Supplemental table S4: predicted target gene of miR-151a-30 by miRDB. Supplemental table S5: primer sequences for methylation-specific PCR (MSP).
