# Supplementary material for: Dysregulated Bone Metabolism Is Related to High Expression of miR-151a-3p in Severe Adolescent Idiopathic Scoliosis
Source: Biomed Res Int. 2020 Sep 26;2020:4243015. doi: 10.1155/2020/4243015 (PMC7537684; doi:10.1155/2020/4243015)
Supplement: Supplementary Materials — Supplemental table S1: candidate miRNA expression profile by miRNA sequencing. Supplemental table S2: summary of the primer sequences for RT-PCR. Supplemental table S3: predicted target gene of miR-151a-30 by TargetScan7.2. Supplemental table S4: predicted target gene of miR-151a-30 by miRDB. Supplemental table S5: primer sequences for methylation-specific PCR (MSP). [file 4243015.f1.zip › Supplementary table 1_candidate miRNAs expression.pdf]

red is pvalue<=0.001,orange is pvalue <=0.01,blue is pvalue<=0.05,green is p

Index	miR_name	miR_seq	pvalue(A
1	hsa-miR-941	CACCCGGCTGTGTGCACATGTGC	#######
2	hsa-miR-505-5p_R+1	GGGAGCCAGGAAGTATTGATGTT	#######
3	hsa-miR-151a-3p	CTAGACTGAAGCTCCTTGAGG	#######
4	hsa-miR-15a-5p_R-1	TAGCAGCACATAATGGTTTGT	#######
5	hsa-miR-148b-5p_L+1R-1	GAAGTTCTGTTATACACTCAGG	#######
6	hsa-miR-361-3p	TCCCCCAGGTGTGATTCTGATTT	#######
7	hsa-miR-362-5p_R-2	AATCCTTGGAACCTAGGTGTGA	#######
8	hsa-miR-1301-3p_R+1	TTGCAGCTGCCTGGGAGTGACTTCT	#######
9	hsa-miR-30c-5p_R-2	TGTAAACATCCTACACTCTCA	#######
10	hsa-miR-30b-5p	TGTAAACATCCTACACTCAGCT	#######
11	hsa-miR-24-2-5p_L+1R-1	GTGCCTACTGAGCTGAAACACA	#######
12	hsa-miR-130b-3p	CAGTGCAATGATGAAAGGGCAT	#######
13	hsa-miR-17-3p	ACTGCAGTGAAGGCACTTGTAG	#######
14	hsa-miR-19b-1-5p_1ss23CA	AGTTTTGCAGGTTTGCATCCAGA	#######
15	PC-3p-1965_2907	ATTCCAACGTTCCCGTGG	#######
16	hsa-miR-29c-5p_R-1	TGACCGATTTCTCCTGGTGTT	#######
17	hsa-miR-328-3p	CTGGCCCTCTCTGCCCTTCCGT	#######
18	hsa-miR-107_R-2	AGCAGCATTGTACAGGGCTAT	#######
19	PC-5p-18146_189	CCAATCACGTTCCCGTGG	#######
20	hsa-miR-18a-5p_R-1	TAAGGTGCATCTAGTGCAGATA	#######
21	hsa-miR-503-5p_R-2_1ss21CA	TAGCAGCGGGAACAGTTCTGA	#######
22	cgr-miR-1839-5p	AAGGTAGATAGAACAGGTCTTGT	#######
23	hsa-miR-92a-3p	TATTGCACTTGTCCCGGCCTGT	#######
24	hsa-miR-196b-5p_R-1	TAGGTAGTTTCCTGTTGTTGG	#######
25	hsa-miR-32-5p_R-1	TATTGCACATTACTAAGTTGC	#######
26	hsa-miR-16-5p	TAGCAGCACGTAAATATTGGCG	#######
27	hsa-miR-744-5p	TGCGGGGCTAGGGCTAACAGCA	#######
28	hsa-let-7d-5p	AGAGGTAGTAGGTTGCATAGTT	#######
29	hsa-miR-324-5p_R-1	CGCATCCCCTAGGGCATTGGTG	#######
30	hsa-mir-216b-p3_2ss11AT17AG	TCACACACTTTCCCGTGG	#######
31	hsa-miR-501-5p_R+1	AATCCTTTGTCCCTGGGTGAGAG	#######
32	hsa-miR-423-3p	AGCTCGGTCTGAGGCCCCTCAGT	#######
Following miRNAs do not show significant difference (p-value<=0.05).			
33	hsa-miR-22-3p	AAGCTGCCAGTTGAAGAACTGT	#######
34	hsa-miR-185-3p_R-1	AGGGGCTGGCTTTCCTCTGGT	#######
35	hsa-miR-2355-3p_L-2R+2	TGTCCTTGCTGTTTGGAGATAA	#######
36	hsa-let-7g-5p	TGAGGTAGTAGTTTGTACAGTT	#######
37	hsa-miR-142-5p_L+2R-2	CCCATAAAGTAGAAAGCACTA	#######
38	hsa-miR-125b-5p	TCCCTGAGACCCTAACTTGTGA	#######
39	hsa-miR-550a-3p_R-1	TGTCTTACTCCCTCAGGCACA	#######
40	cgr-miR-1839-3p_R-1	AGACCTACTTATCTACCAACA	#######
41	hsa-miR-126-5p	CATTATTACTTTTGGTACGCG	#######
42	mmu-let-7j_R-2	TGAGGTATTAGTTTGTGCTGTT	#######
43	hsa-miR-185-5p	TGGAGAGAAAGGCAGTTCCTGA	#######
44	hsa-miR-942-5p_R+1	TCTTCTCTGTTTTGGCCATGTGA	#######
45	hsa-miR-409-3p	GAATGTTGCTCGGTGAACCCCT	#######
46	hsa-miR-17-5p	CAAAGTGCTTACAGTGCAGGTAG	#######
47	hsa-miR-106a-5p_1ss1AC	CAAAGTGCTTACAGTGCAGGTAG	#######

48	hsa-miR-182-5p_R-2	TTTGGCAATGGTAGAACTCACA	#######
49	hsa-miR-664a-3p	TATTCATTTATCCCCAGCCTACA	#######
50	hsa-miR-4433b-5p_R+1	ATGTCCCACCCCCACTCCTGTT	#######
51	hsa-miR-93-5p	CAAAGTGCTGTTCGTGCAGGTAG	#######
52	hsa-miR-210-3p	CTGTGCGTGTGACAGCGGCTGA	#######
53	hsa-miR-223-5p_R+1	CGTGTATTTGACAAGCTGAGTTG	#######
54	PC-5p-15457_231	TCCCCCACTGTCCCGTGG	#######
55	hsa-miR-628-5p	ATGCTGACATATTTACTAGAGG	#######
56	hsa-miR-190a-5p_R-1	TGATATGTTTGATATATTAGG	#######
57	hsa-miR-651-5p_R-1	TTTAGGATAAGCTTGACTTTT	#######
58	hsa-miR-10b-5p_R-1	TACCCTGTAGAACCGAATTTGT	#######
59	hsa-miR-100-5p_R-1	AACCCGTAGATCCGAACTTGT	#######
60	hsa-miR-3158-3p_R-1	AAGGGCTTCCTCTCTGCAGGA	#######
61	hsa-miR-101-3p_R+1	TACAGTACTGTGATAACTGAAG	#######
62	hsa-mir-106a-p3	TACTGCAATGTAAGCACTTCT	#######
63	hsa-miR-28-3p	CACTAGATTGTGAGCTCCTGGA	#######
64	hsa-miR-144-3p	TACAGTATAGATGATGTACT	#######
65	hsa-miR-151a-5p	TCGAGGAGCTCACAGTCTAGT	#######
66	hsa-miR-126-3p	TCGTACCGTGAGTAATAATGCG	#######
67	hsa-miR-511-5p	GTGTCTTTTGCTCTGCAGTCA	#######
68	hsa-miR-7-1-3p	CAACAAATCACAGTCTGCCATA	#######
69	cgr-miR-486-5p_R+2	TCCTGTACTGAGCTGCCCCGAGGT	#######
70	hsa-miR-27a-3p_R-1	TTCACAGTGGCTAAGTTCCG	#######
71	hsa-miR-103a-3p	AGCAGCATTGTACAGGGCTATGA	#######
72	mdo-miR-22-3p	AAGCTGCCAGTTGAAGAACTGC	#######
73	hsa-miR-25-3p	CATTGCACTTGTCTCGGTCTGA	#######
74	hsa-miR-628-3p_R+2	TCTAGTAAGAGTGGCAGTCGAAG	#######
75	hsa-miR-139-5p	TCTACAGTGCACGTGTCTCCAGT	#######
76	hsa-miR-450a-5p	TTTTGCGATGTGTTCCTAATAT	#######
77	hsa-let-7i-5p	TGAGGTAGTAGTTTGTGCTGTT	#######
78	hsa-miR-618	AAACTCTACTTGTCCTTCTGAGT	#######
79	hsa-miR-374a-3p	CTTATCAGATTGTATTGTAATT	#######
80	hsa-miR-146b-5p_R+2	TGAGAACTGAATTCCATAGGCTGT	#######
81	hsa-miR-30e-3p_1ss22CT	CTTTCAGTCGGATGTTTACAGT	#######
82	hsa-miR-2110_R+1	TTGGGGAAACGGCCGCTGAGTGA	#######
83	hsa-miR-625-5p_R+1	AGGGGGAAAGTTCTATAGTCCT	#######
84	hsa-miR-548e-3p	AAAAACTGAGACTACTTTTGCA	#######
85	hsa-miR-877-5p_R+2	GTAGAGGAGATGGCGCAGGGGA	#######
86	hsa-miR-543_R+1	AAACATTCGCGGTGCACTTCTTT	#######
87	hsa-miR-99b-5p	CACCCGTAGAACCGACCTTGCG	#######
88	hsa-miR-183-5p	TATGGCACTGGTAGAATTCACT	#######
89	hsa-miR-214-5p	TGCCTGTCTACACTTGCTGTGC	#######
90	hsa-miR-335-5p_R-1	TCAAGAGCAATAACGAAAAATG	#######
91	hsa-miR-484_R-1	TCAGGCTCAGTCCCCTCCCGA	#######
92	hsa-miR-141-3p_R-1	TAACACTGTCTGGTAAAGATG	#######
93	hsa-let-7b-5p	TGAGGTAGTAGGTTGTGTGGTT	#######
94	hsa-miR-532-5p	CATGCCTTGAGTGTAGGACCGT	#######
95	hsa-miR-5010-5p_R-1	AGGGGGATGGCAGAGCAAAAT	#######
96	hsa-miR-106b-5p_R-1	TAAAGTGCTGACAGTGCAGA	#######
97	hsa-miR-29a-3p	TAGCACCATCTGAAATCGGTTA	#######

98	hsa-miR-199a-3p	ACAGTAGTCTGCACATTGGTTA	#######
99	hsa-miR-181a-5p_R-1	AACATTCAACGCTGTCGGTGAG	#######
100	hsa-miR-374a-5p_R+1	TTATAATACAACCTGATAAGTGA	#######
101	hsa-miR-301a-3p	CAGTGCAATAGTATTGTCAAAGC	#######
102	hsa-miR-222-3p	AGCTACATCTGGCTACTGGGT	#######
103	hsa-miR-3200-5p_R+1	AATCTGAGAAGGCGCACAAGGTT	#######
104	hsa-miR-4732-5p	TGTAGAGCAGGGAGCAGGAAGCT	#######
105	hsa-let-7f-5p	TGAGGTAGTAGATTGTATAGTT	#######
106	hsa-miR-34a-5p	TGGCAGTGTCTTAGCTGGTTGT	#######
107	hsa-miR-7641_L+4R+1_1ss5TC	TCGTCTGATCTCGGAAGCTAAGCA	#######
108	hsa-miR-29b-3p_R-2	TAGCACCATTTGAAATCAGTG	#######
109	hsa-miR-20a-5p_R-1	TAAAGTGCTTATAGTGCAGGTA	#######
110	hsa-miR-144-5p	GGATATCATCATATACTGTAAG	#######
111	hsa-miR-150-5p	TCTCCCAACCCTTGTACCAGTG	#######
112	hsa-miR-205-5p_R-1	TCCTTCATTCCACCGGAGTCT	#######
113	hsa-miR-1180-3p	TTTCCGGCTCGCGTGGGTGTGT	#######
114	hsa-miR-200c-3p	TAATACTGCCGGGTAATGATGGA	#######
115	hsa-miR-223-3p_R+1	TGTCAGTTTGTCAAATACCCCAA	#######
116	hsa-miR-145-3p	GGATTCCTGGAAATACTGTTCT	#######
117	hsa-miR-425-5p	AATGACACGATCACTCCCGTTGA	#######
118	hsa-miR-133a-3p_L-1R+1	TTGGTCCCCTTCAACCAGCTGT	#######
119	hsa-miR-375	TTTGTTCGTTCGGCTCGCGTGA	#######
120	hsa-miR-502-3p	AATGCACCTGGGCAAGGATTCA	#######
121	hsa-miR-101-5p_L+1R-2	TCAGTTATCACAGTGCTGATG	#######
122	hsa-miR-363-3p_R-1	AATTGCACGGTATCCATCTGT	#######
123	hsa-miR-128-3p	TCACAGTGAACCGGTCTCTTT	#######
124	hsa-miR-542-3p	TGTGACAGATTGATAACTGAAA	#######
125	hsa-miR-21-5p	TAGCTTATCAGACTGATGTTGA	#######
126	hsa-miR-96-5p_R-2	TTTGGCACTAGCACATTTTTG	#######
127	hsa-miR-203a-3p	GTGAAATGTTTAGGACCACTAG	#######
128	hsa-miR-146a-5p	TGAGAACTGAATTCCATGGGTT	#######
129	hsa-miR-92b-3p_R-2	TATTGCACTCGTCCCGGCCT	#######
130	bta-miR-16b_L+1R+1	GTAGCAGCACGTAAATATTGGCG	#######
131	hsa-miR-18a-3p_R-2	ACTGCCCTAAGTGCTCCTTCT	#######
132	hsa-miR-1226-3p	TCACCAGCCCTGTGTTCCCTAG	#######
133	hsa-miR-4732-3p	GCCCTGACCTGTCCTGTTCTG	#######
134	hsa-miR-29c-3p_R-1	TAGCACCATTTGAAATCGGTT	#######
135	hsa-miR-1307-3p_R+1	ACTCGGCGTGGCGTCGGTCGTGG	#######
136	hsa-miR-20a-3p_R+1	ACTGCATTATGAGCACTTAAAGT	#######
137	hsa-miR-1255b-5p	CGGATGAGCAAAGAAAGTGGTT	#######
138	hsa-miR-10a-5p_R-1	TACCCTGTAGATCCGAATTTGT	#######
139	hsa-miR-1260a_R+3_1ss9TG	ATCCCACCGCTGCCACCAAAA	#######
140	hsa-let-7f-1-3p_1ss22CT	CTATACAATCTATTGCCTTCCT	#######
141	hsa-miR-338-3p_R+1	TCCAGCATCAGTGATTTTGTTGA	#######
142	hsa-miR-874-3p	CTGCCCTGGCCCGAGGGACCGA	#######
143	hsa-miR-660-5p	TACCCATTGCATATCGGAGTTG	#######
144	mdo-miR-106-5p_R-1	AAAAGTGCTTATAGTGCAGGTA	#######
145	hsa-miR-98-5p	TGAGGTAGTAAGTTGTATTGTT	#######
146	hsa-miR-200a-3p_R+1	TAACACTGTCTGGTAACGATGTT	#######
147	hsa-miR-378c_R-5	ACTGGACTTGGAGTCAGAAG	#######

148	hsa-miR-425-3p_L+1R-1	CATCGGGAATGTCGTGTCCGCC	#######
149	hsa-miR-15b-3p_R-1	CGAATCATTATTTGCTGCTCT	#######
150	hsa-miR-532-3p	CCTCCCACACCCAAGGCTTGCA	#######
151	hsa-miR-31-5p_R+2	AGGCAAGATGCTGGCATAGCTGT	#######
152	hsa-miR-1306-5p_R-1	CCACCTCCCCTGCAAACGTCC	#######
153	hsa-miR-191-5p_R-1	CAACGGAATCCCAAAAGCAGCT	#######
154	hsa-miR-200b-3p	TAATACTGCCTGGTAATGATGA	#######
155	hsa-miR-324-3p_R+1	ACTGCCCCAGGTGCTGCTGGT	#######
156	hsa-miR-145-5p	GTCCAGTTTTCCCAGGAATCCCT	#######
157	hsa-let-7i-3p_R-1	CTGCGCAAGCTACTGCCTTGC	#######
158	hsa-miR-495-3p_R+1	AAACAAACATGGTGCACTTCTTT	#######
159	hsa-let-7b-3p_R-1	CTATACAACCTACTGCCTTCC	#######
160	hsa-miR-215-5p_R+1	ATGACCTATGAATTGACAGACA	#######
161	hsa-miR-589-5p_R-1	TGAGAACCACGTCTGCTCTGA	#######
162	hsa-miR-27b-3p	TTCACAGTGGCTAAGTTCTGC	#######
163	hsa-miR-106b-3p_R-2	CCGCACTGTGGGTACTTGCT	#######
164	hsa-miR-429	TAATACTGTCTGGTAAAACCGT	#######
165	hsa-miR-135b-5p	TATGGCTTTTCATTCCTATGTGA	#######
166	hsa-miR-340-5p_1ss5AC	TTATCAAGCAATGAGACTGATT	#######
167	hsa-miR-103a-2-5p_R+1	AGCTTCTTTACAGTGCTGCCTTGA	#######
168	hsa-miR-122-3p_R-1	AACGCCATTATCACACTAAAT	#######
169	hsa-miR-26b-5p_R+1	TTCAAGTAATTCAGGATAGGTT	#######
170	hsa-miR-497-5p	CAGCAGCACACTGTGGTTTGT	#######
171	hsa-miR-148a-3p	TCAGTGCACTACAGAACTTTGT	#######
172	hsa-miR-181d-5p_R+1	AACATTCATTGTTGTCGGTGGGTT	#######
173	hsa-miR-127-3p	TCGGATCCGTCTGAGCTTGGCT	#######
174	hsa-miR-21-3p	CAACACCAGTCGATGGGCTGT	#######
175	hsa-miR-1537-5p	AGCTGTAATTAGTCAGTTTTCT	#######
176	hsa-miR-140-3p_L-1R+1	ACCACAGGGTAGAACCACGGA	#######
177	hsa-miR-500a-3p_L+1R-3	AATGCACCTGGGCAAGGATT	#######
178	hsa-miR-501-3p	AATGCACCCGGGCAAGGATTCT	#######
179	hsa-miR-194-5p	TGTAACAGCAACTCCATGTGGA	#######
180	hsa-miR-31-3p_R+1	TGCTATGCCAACATATTGCCATC	#######
181	hsa-miR-301b-3p	CAGTGCAATGATATTGTCAAAGC	#######
182	hsa-miR-629-5p	TGGGTTTACGTTGGGAGAACT	#######
183	hsa-miR-34c-5p	AGGCAGTGTAGTTAGCTGATTGC	#######
184	hsa-miR-493-5p	TTGTACATGGTAGGCTTTCATT	#######
185	hsa-miR-20b-5p_R-1	CAAAGTGCTCATAGTGCAGGTA	#######
186	hsa-miR-590-5p	GAGCTTATTCATAAAAGTGCAG	#######
187	hsa-miR-326_R+1	CCTCTGGGCCCTTCCTCCAGT	#######
188	hsa-miR-192-5p	CTGACCTATGAATTGACAGCC	#######
189	hsa-miR-1307-5p	TCGACCGGACCTCGACCGGCT	#######
190	hsa-miR-625-3p	GACTATAGAACTTTCCCCCTCA	#######
191	hsa-miR-143-5p_R-1	GGTGCAGTGCTGCATCTCTGG	#######
192	hsa-miR-627-5p	GTGAGTCTCTAAGAAAAGAGGA	#######
193	hsa-miR-193b-5p	CGGGGTTTTGAGGGCGAGATGA	#######
194	hsa-miR-362-3p	AACACACCTATTCAAGGATTCA	#######
195	hsa-miR-199a-5p	CCCAGTGTTCAGACTACCTGTTC	#######
196	hsa-miR-134-5p	TGTGACTGGTTGACCAGAGGGG	#######
197	hsa-let-7a-5p_R-1	TGAGGTAGTAGGTTGTATAGT	#######

198	hsa-miR-3615_R+1	TCTCTCGGCTCCTCGCGGCTCG	#######
199	hsa-miR-18b-5p_R-2	TAAGGTGCATCTAGTGCAGTT	#######
200	hsa-miR-10a-3p_R-1	CAAATTCGTATCTAGGGGAAT	#######
201	hsa-miR-337-5p	GAACGGCTTCATACAGGAGTT	#######
202	hsa-miR-450b-5p_R-1	TTTTGCAATATGTTCCTGAAT	#######
203	hsa-miR-500b-5p_L+1R+4	TAATCCTTGCTACCTGGGTGAGA	#######
204	hsa-miR-30e-5p_R+2	TGTAAACATCCTTGACTGGAAGCT	#######
205	hsa-miR-143-3p_R+1	TGAGATGAAGCACTGTAGCTCT	#######
206	hsa-miR-424-3p	CAAAACGTGAGGCGCTGCTAT	#######
207	hsa-mir-451a-p3	TTTAGTAATGGTAATGGTTCT	#######
208	hsa-miR-483-5p_R+1_1ss22GA	AAGACGGGAGGAAAGAAGGGAAA	#######
209	hsa-miR-548q_L-1R+2	CTGGTGCAAAAGTAATGGCGGTT	#######
210	hsa-miR-4508_L+2R-1	AAGCGGGGCTGGGCGCGC	#######
211	hsa-miR-376c-3p	AACATAGAGGAAATTCCACGT	#######
212	hsa-let-7f-2-3p_1ss22CT	CTATACAGTCTACTGTCTTTCT	#######
213	hsa-miR-29b-2-5p_R+1	CTGGTTTCACATGGTGGCTTAGA	#######
214	hsa-miR-136-3p	CATCATCGTCTCAAATGAGTCT	#######
215	hsa-miR-221-5p	ACCTGGCATACAATGTAGATTT	#######
216	hsa-miR-296-5p	AGGGCCCCCCCTCAATCCTGT	#######
217	hsa-miR-125a-5p_R-2	TCCCTGAGACCCTTTAACCTGT	#######
218	hsa-miR-381-3p	TATACAAGGGCAAGCTCTCTGT	#######
219	hsa-miR-4772-5p	TGATCAGGCAAAATTGCAGACT	#######
220	hsa-miR-660-3p_R+2	ACCTCCTGTGTGCATGGATTACA	#######
221	hsa-miR-483-3p_L-1R+2	CACTCCTCTCCTCCCGTCTTCT	#######
222	hsa-miR-193b-3p	AACTGGCCCTCAAAGTCCCGCT	#######
223	hsa-miR-199b-5p_R-1	CCCAGTGTTTAGACTATCTGTT	#######
224	hsa-miR-99a-5p_R-1	AACCCGTAGATCCGATCTTGT	#######
225	hsa-miR-624-5p	TAGTACCAGTACCTTGTGTTCA	#######
226	hsa-miR-376a-3p_1ss6AG	ATCATGGAGGAAAATCCACGT	#######
227	ptr-miR-320d_R+4	AAAAGCTGGGTTGAGAGGGAAA	#######
228	mml-miR-320b_1ss20CA	AAAAGCTGGGTTGAGAGGGAAA	#######
229	hsa-miR-331-3p	GCCCCTGGGCCTATCCTAGAA	#######
230	hsa-miR-10b-3p_L-2	AGATTCGATTCTAGGGGAAT	#######
231	hsa-miR-190b_R+1	TGATATGTTTGATATTGGGTTG	#######
232	hsa-miR-7-5p	TGGAAGACTAGTGATTTTGTTGT	#######
233	hsa-miR-487a-3p	AATCATACAGGGACATCCAGTT	#######
234	hsa-miR-19a-3p	TGTGCAAATCTATGCAAAACTGA	#######
235	hsa-miR-122-5p	TGGAGTGTGACAATGGTGTTTG	#######
236	hsa-miR-433-3p	ATCATGATGGGCTCCTCGGTGT	#######
237	hsa-miR-197-3p	TTCACCACCTTCTCCACCCAGC	#######
238	hsa-miR-329-3p_L-1R+1	ACACACCTGGTTAACCTCTTTA	#######
239	hsa-miR-1228-5p	GTGGGCGGGGGCAGGTGTGTG	#######
240	hsa-miR-675-3p_L-1R+2	TGTATGCCCTCACCGCTCAGC	#######
241	hsa-miR-3940-3p_R-1	CAGCCCGGATCCCAGCCCACT	#######
242	hsa-miR-598-3p	TACGTCATCGTTGTCATCGTCA	#######
243	hsa-miR-337-3p_L-1	TCCTATATGATGCCTTTCTTC	#######
244	hsa-miR-579-3p	TTCATTTGGTATAAACCGCGATT	#######
245	hsa-miR-320c_R-1	AAAAGCTGGGTTGAGAGGG	#######
246	hsa-miR-193a-5p	TGGGTCTTTGCGGGCGAGATGA	#######
247	hsa-miR-152-3p	TCAGTGCATGACAGAACTTGG	#######

248	hsa-miR-148b-3p	TCAGTGCATCACAGAACTTTGT	#######
249	hsa-miR-204-5p	TTCCCTTTGTCATCCTATGCCT	#######
250	hsa-mir-3656-p3_1ss17AC	CGGGTGCGGGGGTGGGCGG	#######
251	hsa-miR-3143_R-4	ATAACATTGTAAAGCGCTTCT	#######
252	hsa-miR-5010-3p	TTTTGTGTCTCCCATTCCCCAG	#######
253	hsa-miR-1908-5p_R-1	CGGCGGGGACGGCGATTGGT	#######
254	hsa-miR-16-2-3p_L+1R-2	ACCAATATTACTGTGCTGCTT	#######
255	hsa-miR-30d-5p	TGTAAACATCCCCGACTGGAAG	#######
256	hsa-miR-26a-5p	TTCAAGTAATCCAGGATAGGCT	#######
257	hsa-miR-582-5p	TTACAGTTGTTCAACCAGTTACT	#######
258	PC-3p-25234_121	GGAGGGCGCGCGGGTCGG	#######
259	hsa-miR-30a-3p_R-1	CTTTCAGTCGGATGTTTGCAG	#######
260	hsa-miR-142-3p_L-1	GTAGTGTTTCCTACTTTATGGA	#######
261	hsa-miR-369-3p	AATAATACATGGTTGATCTTT	#######
262	hsa-miR-221-3p_R-1	AGCTACATTGTCTGCTGGGTTT	#######
263	hsa-miR-188-5p_R+1	CATCCCTTGCATGGTGGAGGGT	#######
264	hsa-miR-140-5p	CAGTGGTTTTACCCTATGGTAG	#######
265	hsa-miR-155-5p	TTAATGCTAATCGTGATAGGGGT	#######
266	hsa-let-7d-3p	CTATACGACCTGCTGCCTTTCT	#######
267	hsa-miR-30a-5p	TGTAAACATCCTCGACTGGAAG	#######
268	hsa-miR-576-5p_R+1	ATTCTAATTTCTCCACGTCTTTG	#######
269	hsa-miR-320d_R-1	AAAAGCTGGGTTGAGAGG	#######
270	hsa-miR-26b-3p_R-1	CCTGTTCTCCATTACTTGGCT	#######
271	hsa-miR-135a-5p	TATGGCTTTTTATTCCTATGTGA	#######
272	hsa-miR-9-3p	ATAAAGCTAGATAACCGAAAGT	#######
273	hsa-miR-195-5p_R+1	TAGCAGCACAGAAATATTGGCA	#######
274	hsa-miR-15b-5p	TAGCAGCACATCATGGTTTACA	#######
275	hsa-miR-320a	AAAAGCTGGGTTGAGAGGGCGA	#######
276	hsa-miR-1260b_R+1_1ss9AG	ATCCCACCGCTGCCACCATT	#######
277	hsa-miR-590-3p	TAATTTTATGTATAAGCTAGT	#######
278	hsa-miR-24-3p	TGGCTCAGTTCAGCAGGAACAG	#######
279	hsa-miR-1-3p	TGGAATGTAAAGAAGTATGTAT	#######
280	hsa-miR-33a-5p_R-1	GTGCATTGTAGTTGCATTGC	#######
281	hsa-miR-486-3p	CGGGGCAGCTCAGTACAGGAT	#######
282	hsa-miR-423-5p	TGAGGGGCAGAGAGCGAGACTTT	#######
283	hsa-miR-424-5p_R-1	CAGCAGCAATTCATGTTTTGA	#######
284	hsa-miR-181b-5p_R+1	AACATTCATTGCTGTCGGTGGGTT	#######
285	hsa-miR-550a-3-5p	AGTGCCTGAGGGAGTAAGAG	#######
286	hsa-miR-550a-5p_R-3	AGTGCCTGAGGGAGTAAGAG	#######
287	hsa-miR-377-3p	ATCACACAAAGGCAACTTTTGT	#######
288	hsa-miR-335-3p	TTTTTCATTATTGCTCCTGACC	#######
289	hsa-miR-93-3p_R+1	ACTGCTGAGCTAGCACTTCCCGA	#######
290	hsa-miR-452-5p	AACTGTTTGCAGAGGAAACTGA	#######
291	hsa-miR-339-3p_R-3	TGAGCGCCTCGACGACAGAG	#######
292	hsa-miR-505-3p	CGTCAACACTTGCTGGTTTCCT	#######
293	hsa-miR-339-5p	TCCCTGTCCTCCAGGAGCTCACG	#######
294	hsa-miR-454-3p	TAGTGCAATATTGCTTATAGGGT	#######
295	hsa-let-7a-3p	CTATACAATCTACTGTCTTTC	#######
296	hsa-miR-338-5p_R-1	AACAATATCCTGGTGCTGAGT	#######
297	hsa-miR-214-3p_R-1	ACAGCAGGCACAGACAGGCAG	#######

298	hsa-miR-1537-3p	AAAACCGTCTAGTTACAGTTGT	#######
299	hsa-miR-766-3p_R-1	ACTCCAGCCCCACAGCCTCAG	#######
300	hsa-miR-486-5p	TCCTGTACTGAGCTGCCCCGAG	#######
301	hsa-miR-23a-3p_R+1	ATCACATTGCCAGGGATTTCCA	#######
302	hsa-miR-361-5p	TTATCAGAATCTCCAGGGGTAC	#######
303	hsa-miR-769-5p	TGAGACCTCTGGGTTCTGAGCT	#######
304	hsa-miR-136-5p_R-1	ACTCCATTTGTTTTGATGATGG	#######
305	hsa-miR-22-5p	AGTTCTTCAGTGGCAAGCTTTA	#######
306	hsa-miR-186-5p	CAAAGAATTCTCCTTTTGGGCT	#######
307	hsa-miR-451a	AAACCGTTACCATTACTGAGTT	#######
308	hsa-let-7e-5p	TGAGGTAGGAGGTTGTATAGTT	#######
309	hsa-miR-382-5p	GAAGTTGTTCGTGGTGGATTCG	#######
310	hsa-miR-487b-3p	AATCGTACAGGGTCATCCACTT	#######
311	hsa-miR-499a-5p	TTAAGACTTGCAGTGATGTTT	#######
312	hsa-miR-365b-3p	TAATGCCCCTAAAAATCCTTAT	#######
313	hsa-miR-33b-5p_R-1	GTGCATTGCTGTTGCATTG	#######
314	hsa-miR-23b-3p_R+1	ATCACATTGCCAGGGATTACCA	#######
315	hsa-miR-19b-3p	TGTGCAAATCCATGCAAAACTGA	#######
316	hsa-miR-494-3p_R+1	TGAAACATACACGGGAAACCTCT	#######

value <=0.1.

AISFU/AI	AISFU/AI	AISFU/AI	AISFU/AI	AISFU/AI	AISO/AISO_	AISO/AIS	AISO/AIS	AISO/AIS
0	5	4	8	5	12	28	19	23
0	5	6	4	0	15	12	19	23
108	50	77	53	23	273	186	258	470
3,628	4,966	3,863	2,588	2,889	2,199	2,956	803	1,428
0	6	18	4	9	13	31	22	13
0	6	6	7	13	14	9	9	12
37	32	63	12	43	39	46	22	32
0	5	3	0	13	12	8	16	5
339	272	419	309	448	482	361	666	650
419	481	638	369	590	770	604	529	620
0	0	9	12	5	43	22	135	58
31	51	47	48	9	32	41	0	25
45	116	85	52	55	25	50	16	19
0	3	3	4	0	4	4	0	5
1,288	227	385	276	183	139	104	76	77
0	0	5	4	0	35	19	46	30
4	9	19	26	0	30	15	22	24
492	745	597	247	247	118	172	50	113
113	6	20	16	0	6	3	0	0
396	507	409	201	312	208	189	290	180
0	6	5	4	9	3	0	0	0
10	42	48	85	19	82	53	63	94
3,935	6,809	2,732	2,552	2,978	1,899	3,177	474	1,378
10	12	6	23	8	12	24	0	17
166	296	135	143	160	140	195	35	73
23,356	37,966	20,885	11,998	20,313	11,094	24,297	2,834	10,135
63	29	62	131	52	62	52	54	120
1,090	1,121	1,731	1,433	814	2,155	1,200	925	1,084
55	69	68	35	39	26	41	16	24
63	17	4	11	5	0	3	0	0
0	8	10	0	5	5	18	25	2
246	184	302	362	431	442	392	1,659	814
5,228	3,608	3,881	3,704	3,695	2,847	3,705	1,095	2,178
0	2	8	4	0	6	8	0	6
0	0	0	4	0	10	0	0	14
4,123	5,193	6,433	3,656	4,920	8,108	6,322	3,106	4,335
978	1,242	1,490	2,426	938	2,669	1,714	1,294	2,531
374	48	167	146	293	864	143	861	560
8	5	6	0	0	6	4	0	3
0	8	2	11	5	8	5	0	0
1,102	800	295	1,555	414	691	339	76	223
0	11	11	8	0	2	3	0	4
1,550	2,458	2,132	1,171	1,500	1,019	1,472	450	853
0	14	9	4	0	15	16	0	14
0	0	0	12	0	3	0	16	12
1,850	3,628	2,464	1,414	1,943	1,003	1,501	993	1,198
1,850	3,628	2,464	1,414	1,943	1,003	1,501	993	1,198

368	569	625	112	897	460	566	651	420
0	4	3	20	13	21	4	22	15
0	6	20	36	16	40	10	35	16
2,431	5,419	2,840	2,410	2,782	1,436	1,696	869	2,013
24	42	41	10	16	6	4	0	12
39	23	24	209	57	37	88	428	322
31	15	9	9	0	29	53	0	0
0	0	2	6	0	14	5	0	8
42	89	103	28	51	26	31	0	24
0	0	0	5	0	3	9	0	3
1,067	147	570	616	2,153	2,692	1,524	9,288	2,270
135	17	119	60	55	215	66	203	187
0	1	1	0	0	1	2	0	0
1,593	1,606	1,694	667	1,764	328	687	1,402	1,078
8	4	4	0	0	4	4	0	0
18	7	21	52	40	29	38	198	70
5,424	9,410	6,630	1,090	1,985	3,959	2,670	79	475
187	114	199	222	104	429	168	149	136
6,262	2,571	3,762	5,215	6,013	6,829	4,605	9,650	4,837
8	0	3	0	5	11	0	19	8
0	0	6	8	6	2	4	0	11
0	3	0	0	0	2	8	0	4
2,052	740	717	2,993	1,164	1,345	700	480	1,244
1,689	3,251	2,619	2,007	2,585	1,412	2,002	2,754	1,737
9	5	4	14	11	5	3	0	2
4,110	4,775	3,884	2,170	2,517	3,037	2,790	406	1,581
0	6	2	14	5	4	4	0	6
108	22	34	112	86	147	33	110	110
4	4	6	24	8	6	7	9	43
2,450	3,747	2,798	2,532	2,384	2,345	2,962	1,869	1,553
0	0	0	7	0	0	7	0	10
0	0	2	0	3	4	0	9	2
32	61	30	182	57	61	88	85	69
44	67	60	156	60	103	61	38	143
8	17	27	12	22	16	24	0	33
8	3	6	17	0	14	19	0	7
0	0	0	4	7	0	6	19	4
0	5	4	0	0	11	5	0	7
0	0	0	14	0	5	3	16	6
68	3	38	47	50	90	37	138	127
45	128	102	14	187	61	81	44	59
0	0	2	7	7	2	4	0	4
131	93	169	330	379	294	363	1,413	572
596	557	786	381	430	850	769	651	606
37	5	24	15	31	9	15	113	22
5,466	9,596	7,012	4,536	3,645	5,445	5,377	2,087	3,136
73	92	54	63	51	50	71	31	36
0	2	5	0	5	6	12	0	2
2,482	2,436	3,575	873	1,261	1,465	1,167	482	939
1,344	644	662	1,450	922	2,136	946	1,939	1,690

284	62	176	489	214	602	154	480	394
276	322	230	348	329	276	233	258	251
10	12	8	35	16	7	15	47	40
27	79	59	55	71	18	51	66	46
370	292	228	706	272	264	214	375	293
0	11	20	0	11	5	30	16	4
8	27	19	0	21	9	13	0	8
1,137	1,292	1,319	1,902	1,010	2,631	1,247	1,054	1,394
13	0	7	20	0	0	3	0	15
0	4	0	0	0	5	8	0	29
92	163	122	100	120	223	179	131	128
2,835	5,473	3,579	1,837	3,726	2,288	3,315	2,222	2,062
207	571	433	200	338	391	302	318	349
958	799	678	603	1,172	2,390	1,271	1,369	575
47	7	13	18	28	6	6	0	2
15	15	22	4	0	12	13	0	0
11	6	41	26	38	7	6	167	95
5,708	6,394	5,184	53,632	7,514	8,374	11,472	34,683	74,405
0	0	13	7	0	48	5	0	37
669	1,029	987	799	583	682	936	570	598
0	1	4	7	0	4	0	9	6
318	23	250	77	522	315	81	142	189
36	28	42	20	40	43	43	25	28
18	28	15	6	9	12	4	0	5
396	584	545	114	320	284	391	47	123
23	25	36	89	15	78	31	42	65
0	0	3	5	0	0	0	0	12
7,274	4,209	7,063	10,621	9,912	6,973	7,209	16,224	7,512
142	395	253	45	423	303	428	145	129
0	0	0	0	0	2	0	0	0
262	196	213	795	226	172	207	129	221
0	4	3	0	0	0	0	0	0
18	33	17	9	26	3	21	19	12
0	21	13	0	7	15	7	0	0
0	2	3	0	0	0	0	0	0
0	14	12	4	0	8	0	0	0
400	287	220	341	271	272	232	378	275
8	6	14	41	7	5	4	0	13
0	8	8	18	12	6	12	35	23
0	2	6	0	0	2	0	0	0
315	61	251	216	593	810	368	2,685	655
0	0	3	0	0	4	0	0	0
0	0	3	0	0	4	0	0	2
13	13	13	103	14	23	12	76	130
0	0	3	0	0	21	5	0	0
145	209	149	123	237	109	178	88	101
21	35	35	12	25	12	20	16	16
81	128	98	214	43	88	60	16	74
32	8	65	7	31	10	10	302	117
37	13	43	10	14	6	6	0	9

0	4	3	28	0	6	8	0	35
68	100	71	44	64	120	71	72	52
24	18	19	13	13	18	15	0	21
0	2	26	0	0	2	0	76	3
0	3	0	4	0	4	0	0	2
1,411	2,821	2,346	4,966	1,810	2,093	2,454	950	3,429
42	9	93	8	60	24	14	362	135
73	91	84	28	59	49	70	22	18
134	60	96	280	144	233	55	1,334	535
29	22	25	23	16	9	16	0	9
0	0	5	23	0	2	3	0	22
0	0	4	10	16	39	4	0	13
86	11	41	10	9	46	23	69	75
0	3	6	4	0	3	5	0	4
562	185	253	858	437	588	198	546	638
191	183	288	81	192	198	223	31	75
0	0	13	0	8	7	3	47	28
0	0	3	0	0	0	0	16	0
0	1	1	14	0	2	4	0	1
8	25	12	23	16	3	20	0	15
0	0	0	4	5	20	0	0	0
1,673	2,984	1,980	3,817	1,671	2,250	1,811	727	1,991
92	13	28	78	35	63	26	0	27
868	505	726	1,275	768	720	839	1,778	1,052
0	2	5	12	0	8	0	0	9
0	0	0	20	0	15	6	0	6
0	0	6	0	0	3	3	0	2
0	0	0	4	0	0	0	0	11
616	475	610	489	486	265	438	598	404
0	9	24	7	15	16	21	50	8
21	16	18	9	19	5	5	0	12
157	73	180	43	105	42	35	161	184
0	0	2	0	0	0	0	0	0
0	0	0	7	0	2	4	0	2
0	28	13	34	0	3	13	0	11
0	0	0	0	0	0	0	16	5
0	0	0	5	0	2	0	0	2
357	763	407	161	441	306	426	236	226
10	22	17	28	26	0	3	31	26
16	4	7	20	5	12	3	0	2
540	178	438	123	320	758	342	947	626
10	0	5	4	0	2	0	0	3
0	0	3	0	0	2	0	0	2
0	0	8	0	0	4	0	0	33
0	6	0	6	0	9	12	0	8
0	0	0	0	0	0	0	0	0
10	11	13	12	6	9	5	0	10
61	20	65	139	81	82	48	386	144
0	0	0	7	0	3	0	0	8
1,529	1,649	1,673	1,895	1,032	2,238	1,151	808	1,245

31	43	27	39	13	33	16	0	16
0	34	29	4	11	14	7	0	0
0	0	0	0	0	2	0	0	0
0	0	0	0	0	2	0	0	0
0	3	0	8	0	2	9	16	4
0	7	5	0	8	7	13	0	3
1,795	1,992	1,562	2,195	2,470	1,469	2,932	2,597	2,711
1,055	263	567	891	234	1,239	121	1,284	3,133
0	5	0	9	0	16	0	0	10
8	6	4	0	0	5	0	0	0
0	0	0	0	0	0	0	0	0
0	0	0	0	0	0	0	0	2
0	0	2	0	0	6	0	0	0
0	0	0	8	0	0	0	0	1
0	0	0	2	0	0	0	0	1
0	3	2	0	0	0	0	0	6
0	0	0	0	0	4	0	0	0
0	0	2	5	0	2	0	0	4
0	3	4	0	5	6	0	0	0
335	72	128	323	309	386	169	431	495
0	0	0	6	0	0	0	0	2
0	0	0	5	0	0	0	0	2
0	0	0	0	0	2	0	0	3
0	1	0	0	0	5	0	0	0
21	3	4	5	8	42	5	0	22
16	15	17	136	31	15	13	145	149
410	54	132	203	145	523	118	231	486
0	25	12	5	5	18	9	0	3
0	0	0	6	0	0	0	0	3
0	8	16	8	7	3	21	0	5
0	8	16	8	7	3	21	0	5
11	18	17	29	11	25	11	0	2
0	0	0	0	0	4	0	0	0
0	0	0	0	0	2	0	0	0
19	28	51	12	69	30	25	50	97
0	0	0	4	0	0	0	0	0
690	1,538	725	534	3,905	432	2,183	7,943	1,990
3,929	469	476	2,073	2,058	4,982	819	4,029	3,429
0	0	0	0	0	2	0	0	0
24	19	28	109	22	58	20	31	124
0	0	0	2	0	3	0	0	0
0	0	3	0	0	3	0	0	0
0	0	0	0	0	6	0	0	0
0	3	0	0	0	4	0	0	0
0	6	6	7	0	2	0	0	2
0	0	0	9	0	6	0	0	0
0	3	0	0	0	2	0	0	0
23	4	17	13	5	26	11	0	10
34	15	27	153	33	54	14	57	153
84	10	41	71	80	67	47	98	61

192	208	315	416	313	175	253	255	261
0	0	0	4	0	0	0	0	0
0	0	3	0	0	3	0	0	0
0	0	2	0	0	2	0	0	0
0	0	2	0	0	0	0	0	0
0	0	0	4	0	0	0	0	0
278	397	302	91	219	275	223	192	126
1,029	1,062	1,141	1,172	1,394	1,447	1,692	1,127	1,179
1,089	1,370	1,083	2,938	1,055	1,295	1,150	814	1,354
0	0	0	35	0	0	0	0	12
10	2	12	9	0	19	5	0	0
13	9	6	24	5	20	3	0	28
2,069	3,101	2,156	7,140	4,546	685	1,515	9,373	5,307
0	3	0	23	0	0	0	0	3
874	472	735	2,064	813	495	477	1,467	982
0	0	0	0	6	5	0	0	0
82	71	52	329	125	43	47	201	215
16	22	19	44	37	41	19	94	26
152	77	97	170	115	178	66	94	104
477	138	151	340	388	334	209	324	297
0	13	6	0	0	5	11	0	14
4	1	2	1	0	4	1	0	0
0	2	3	4	0	6	0	0	2
0	2	0	0	0	1	2	0	1
0	0	0	3	0	2	0	0	0
44	16	16	91	20	76	14	35	44
2,524	2,973	2,782	1,955	2,085	3,339	1,935	2,024	1,852
3,550	3,000	4,095	2,925	3,506	2,527	3,789	6,496	2,904
0	0	4	0	0	7	0	0	0
16	42	11	67	22	39	27	0	34
916	481	525	2,605	790	840	567	768	1,538
7	0	9	22	3	3	5	31	11
31	4	27	28	19	16	10	0	7
39	45	38	18	19	49	36	0	10
957	1,467	978	1,396	1,039	937	1,187	1,020	1,078
299	298	201	782	286	223	264	198	489
32	15	16	36	36	38	14	44	33
0	3	2	0	0	0	1	0	0
0	3	2	0	0	0	1	0	0
0	0	3	12	5	7	0	0	6
0	0	0	6	0	2	0	0	12
27	34	37	32	26	30	37	19	56
0	0	0	0	9	2	0	0	5
10	11	29	9	0	25	15	0	11
8	6	27	40	12	34	12	16	41
8	5	16	12	0	13	0	16	12
47	199	59	115	180	99	137	31	125
6	6	2	5	3	11	0	0	1
8	3	5	21	7	9	0	0	14
61	4	10	26	13	88	0	0	24

0	3	3	9	0	2	0	0	12
0	0	2	17	7	6	3	0	0
7,373	9,252	9,209	2,164	7,371	8,542	12,376	4,916	3,963
1,874	660	836	3,878	1,434	1,233	588	3,610	3,087
19	19	13	81	14	24	14	9	34
0	3	5	16	0	7	3	0	2
0	3	2	10	0	5	0	0	3
92	50	67	128	66	80	52	132	92
186	317	245	495	378	269	585	249	356
297,287	368,731	334,038	67,703	294,069	241,800	424,983	278,909	150,369
32	31	40	119	40	89	31	45	74
8	0	5	12	0	6	0	0	5
0	0	0	15	0	2	0	0	2
13	0	4	8	0	6	5	0	2
11	4	4	46	4	19	6	0	48
0	0	2	0	0	0	3	0	0
124	39	42	310	51	117	29	142	200
1,275	3,058	1,301	1,029	5,485	475	2,795	4,797	2,775
0	0	0	11	0	0	3	0	5

AISO/AIS	NC/NC_1(	NC/NC_2(	NC/NC_3(	NC/NC_4(	NC/NC_5(	Expression level
28	0	0	0	0	0	middle
11	0	0	0	0	3	middle
511	43	19	40	45	20	middle
2,738	6,220	3,777	4,179	3,975	4,501	high
10	0	0	0	9	3	middle
11	0	4	0	4	6	middle
28	8	11	4	17	13	middle
4	0	0	0	0	0	middle
372	242	239	373	293	180	middle
548	430	395	503	287	226	middle
31	0	0	0	0	3	middle
41	68	54	68	44	97	middle
51	59	105	106	68	64	middle
2	0	0	0	0	0	low
355	1,222	931	1,633	440	601	middle
10	0	0	0	0	0	middle
23	0	18	0	0	10	middle
381	644	551	308	328	510	middle
8	235	139	186	26	26	middle
266	674	373	553	436	256	middle
6	0	0	0	0	0	low
48	0	25	0	7	62	middle
2,735	4,454	3,584	3,850	4,286	4,411	high
10	0	7	0	0	6	middle
136	299	186	205	189	275	middle
17,641	34,696	19,275	32,588	39,322	22,389	high
89	27	33	0	36	25	middle
1,253	902	717	683	678	621	middle
41	63	56	37	32	48	middle
4	186	79	137	17	174	middle
6	0	0	0	0	0	middle
362	226	200	124	243	149	middle
4,079	4,871	4,696	3,682	3,964	8,149	high
3	0	0	0	0	0	low
6	0	0	0	0	0	middle
5,260	3,626	3,290	3,099	3,850	2,599	high
1,791	1,236	1,589	714	906	1,308	high
174	129	286	211	105	280	middle
2	0	0	0	0	0	low
5	0	0	0	0	0	middle
560	1,231	1,573	615	530	1,303	middle
8	0	0	0	0	0	middle
1,270	1,344	1,040	1,403	2,206	1,097	high
4	0	0	0	0	4	middle
8	0	0	0	0	0	middle
1,832	2,557	1,889	2,232	3,080	1,225	high
1,832	2,557	1,889	2,232	3,080	1,225	high

477	158	192	230	475	186	middle
4	0	0	0	9	0	middle
23	0	12	0	0	17	middle
2,601	3,255	2,174	3,608	4,704	1,841	high
13	63	44	0	12	45	middle
78	0	25	0	57	40	middle
4	163	83	106	7	0	middle
0	0	0	0	0	0	middle
52	54	28	0	55	31	middle
2	0	0	0	0	0	low
1,007	398	385	478	925	801	high
83	177	227	211	34	368	middle
2	0	0	0	0	0	low
1,252	1,492	1,382	1,307	1,699	1,139	middle
4	0	0	0	0	0	low
38	23	11	16	19	15	middle
2,876	9,388	6,227	4,080	2,813	5,689	high
211	93	165	96	73	94	middle
4,443	2,725	5,174	3,602	3,830	3,004	high
4	0	0	0	0	8	middle
4	0	0	0	0	0	middle
0	0	0	0	0	0	low
1,568	2,118	2,729	1,894	901	4,038	high
1,830	1,814	1,436	1,976	2,206	1,343	high
10	29	12	0	12	14	middle
3,118	3,793	2,663	3,217	3,279	2,617	high
2	0	0	0	0	3	middle
82	0	86	0	52	62	middle
15	0	0	0	0	7	middle
2,538	2,254	2,188	2,062	2,444	2,098	high
4	0	0	0	0	0	low
0	0	0	0	0	0	low
50	217	91	186	105	68	middle
88	32	44	0	63	55	middle
6	0	0	0	15	11	middle
4	0	0	0	0	6	middle
2	0	0	0	0	0	middle
0	0	0	0	0	3	middle
2	0	0	0	0	0	middle
56	86	74	124	15	64	middle
87	0	0	0	106	46	middle
13	0	0	0	0	0	middle
253	113	165	149	337	517	middle
424	652	483	391	385	450	middle
33	167	39	621	12	119	middle
4,515	5,708	5,622	5,154	5,985	5,905	high
60	77	65	50	63	100	middle
0	0	0	0	0	0	middle
1,862	2,204	2,049	1,049	1,476	1,562	high
1,247	1,516	1,593	1,580	640	2,589	high

376	279	295	292	114	293	middle
320	199	275	261	251	284	middle
18	0	0	16	24	5	middle
38	0	28	0	86	20	middle
310	1,276	408	453	232	651	middle
14	0	0	0	15	0	middle
6	0	0	0	10	17	middle
1,299	1,279	968	1,217	831	690	high
16	32	32	0	0	45	middle
0	0	0	0	0	4	middle
124	201	134	134	136	159	middle
2,662	4,691	2,979	3,471	5,743	2,298	high
229	249	213	155	305	237	middle
1,099	1,041	1,251	758	1,182	1,029	high
22	23	74	0	9	144	middle
10	0	0	0	0	12	middle
43	258	62	720	23	39	middle
21,051	4,830	7,728	4,601	9,718	7,097	high
12	0	11	0	0	32	middle
717	693	571	553	727	690	middle
3	0	0	0	4	2	low
106	127	576	528	67	708	middle
26	0	22	0	34	43	middle
13	32	14	0	15	18	middle
334	435	297	261	396	379	middle
48	0	51	0	34	43	middle
8	0	0	0	0	0	middle
9,198	13,186	9,979	19,877	6,278	11,503	high
239	122	84	106	233	122	middle
0	0	9	75	0	6	middle
324	290	431	298	310	698	middle
3	23	0	0	0	10	middle
14	32	0	56	70	9	middle
14	0	0	0	0	7	middle
2	0	0	0	0	0	low
4	0	0	0	0	6	middle
291	321	521	233	270	755	middle
16	0	0	0	0	21	middle
8	0	9	0	23	3	middle
0	0	0	0	0	0	low
317	1,575	318	1,360	262	418	high
2	0	0	0	0	0	low
0	0	0	0	0	0	low
53	23	12	37	19	26	middle
4	0	11	0	9	12	middle
156	113	139	168	198	413	middle
17	23	25	0	34	6	middle
112	95	72	62	86	97	middle
71	285	46	1,602	16	83	middle
24	36	26	0	0	45	middle

19	0	11	0	0	3	middle
72	104	33	0	54	57	middle
16	0	0	0	0	35	middle
0	724	0	99	0	12	middle
0	0	0	0	0	0	low
2,274	1,874	1,452	1,894	2,117	1,256	high
127	263	107	1,739	15	70	middle
56	32	72	0	58	76	middle
91	199	142	534	229	261	middle
19	23	39	0	0	60	middle
8	0	0	0	0	0	middle
14	0	0	0	0	21	middle
25	45	56	655	10	82	middle
2	0	0	0	0	4	low
453	444	658	758	332	1,397	middle
161	100	158	68	159	152	middle
11	27	12	279	0	15	middle
4	0	12	50	0	0	middle
6	0	0	0	0	0	middle
8	0	0	0	31	12	middle
4	0	11	0	7	38	middle
2,202	2,616	1,666	2,018	1,824	2,123	high
55	50	84	43	12	213	middle
1,189	751	889	1,757	739	1,934	middle
6	0	0	0	0	6	middle
3	0	0	0	0	3	middle
6	0	21	0	0	10	middle
2	0	0	0	0	0	middle
492	471	408	379	691	512	middle
16	0	17	0	0	38	middle
13	0	21	0	9	44	middle
119	102	96	1,108	61	183	middle
0	45	0	0	0	0	middle
0	0	0	0	0	0	low
12	0	9	0	10	15	middle
0	0	0	0	0	10	middle
2	0	0	0	0	0	low
326	406	365	302	669	337	middle
19	36	21	0	70	24	middle
8	0	19	0	7	6	middle
368	407	415	3,921	194	492	high
3	0	11	0	0	21	middle
3	0	0	0	0	3	low
2	0	0	0	0	8	middle
3	0	0	0	7	12	middle
2	0	0	0	0	13	middle
12	0	26	0	16	26	middle
94	138	120	267	34	55	middle
0	0	0	0	0	0	low
1,298	1,572	1,326	1,414	981	956	high

32	0	32	0	20	47	middle
14	0	12	0	13	22	middle
0	0	0	0	0	8	low
0	0	0	0	0	8	low
2	0	0	0	0	16	middle
3	0	0	0	4	6	middle
1,801	1,276	1,301	1,546	2,954	2,200	high
561	1,064	1,121	3,875	319	440	high
3	0	0	0	0	10	middle
2	0	0	0	0	8	low
2	0	0	0	0	5	low
0	0	0	0	7	0	low
0	0	0	0	0	0	low
2	0	0	0	0	0	low
1	0	0	0	0	0	low
0	0	0	0	0	0	low
0	0	0	0	0	10	low
3	27	0	0	0	3	middle
0	0	0	0	0	4	low
175	267	292	491	160	208	middle
2	0	0	0	0	0	low
0	0	0	0	0	0	low
0	0	0	0	0	6	low
2	0	0	0	0	20	middle
13	0	12	0	0	29	middle
55	50	44	81	19	32	middle
188	186	318	236	73	767	middle
6	0	0	0	10	10	middle
0	0	0	0	0	0	low
10	0	0	0	5	14	middle
10	0	0	0	5	14	middle
18	23	9	0	9	23	middle
0	0	0	0	0	7	low
0	0	0	0	0	4	low
36	33	4	48	53	19	middle
2	0	0	0	0	0	low
527	625	308	528	4,399	371	high
2,253	684	3,163	702	1,928	14,168	high
0	0	0	0	0	3	low
45	23	44	0	23	72	middle
0	0	0	0	0	0	low
2	0	0	0	0	15	middle
0	0	0	0	0	4	low
0	0	0	0	0	0	low
6	0	9	0	0	0	low
0	0	0	0	0	0	low
0	0	0	0	0	0	low
21	12	8	10	5	97	middle
76	0	42	37	20	97	middle
103	50	77	43	41	106	middle

298	222	223	391	173	213	middle
0	0	0	0	0	4	low
0	0	0	0	0	0	low
0	0	0	0	0	0	low
2	0	0	0	0	0	low
0	0	0	0	0	4	low
236	217	199	137	287	257	middle
1,028	828	949	807	1,317	1,728	middle
1,332	1,428	1,350	1,788	1,117	963	high
5	0	0	0	0	6	middle
13	0	21	0	0	48	middle
15	0	9	0	0	27	middle
2,981	2,485	2,670	2,397	4,273	1,530	high
6	0	0	0	0	10	middle
983	1,747	1,107	869	657	1,395	middle
0	0	0	0	0	17	middle
110	77	72	50	181	54	middle
24	68	37	0	28	26	middle
145	50	137	0	67	210	middle
271	163	281	273	246	997	middle
5	0	0	0	16	7	middle
1	0	0	0	0	16	middle
0	0	0	0	0	4	low
1	0	0	0	6	0	low
1	0	0	0	0	8	low
29	0	74	93	29	61	middle
1,912	3,160	1,953	2,099	2,257	2,119	high
2,860	2,515	2,886	2,339	4,018	4,195	high
0	0	0	0	0	13	middle
22	27	26	50	26	26	middle
1,001	659	886	845	804	860	high
6	0	4	19	4	9	middle
41	36	35	0	0	24	middle
25	34	50	0	19	34	middle
868	810	866	565	1,511	1,961	middle
441	204	413	286	310	874	middle
26	11	31	19	22	48	middle
1	0	0	0	0	4	low
1	0	0	0	0	4	low
4	0	0	0	19	9	middle
0	0	9	0	0	0	middle
39	59	21	43	25	16	middle
2	0	0	0	0	4	low
10	0	23	0	9	12	middle
23	0	33	0	7	57	middle
16	0	19	0	0	23	middle
83	59	39	99	255	39	middle
6	0	9	0	0	5	middle
15	0	0	0	0	27	middle
21	32	21	31	16	63	middle

5	0	11	0	0	0	middle
7	0	9	0	0	10	middle
4,483	6,260	5,069	6,073	8,351	5,115	high
1,830	1,718	1,873	1,726	1,814	3,051	high
34	23	30	16	17	61	middle
6	0	11	0	0	5	middle
6	0	11	0	0	9	middle
100	77	81	37	41	174	middle
263	258	195	435	449	206	middle
188,400	404,481	239,300	196,071	428,172	172,667	high
38	50	84	62	28	13	middle
9	0	11	0	7	6	middle
7	0	11	0	7	0	middle
7	0	0	0	0	24	middle
8	0	12	16	7	32	middle
0	0	0	0	0	3	low
88	38	146	149	85	184	middle
967	1,215	603	941	7,874	657	high
3	0	0	0	12	0	middle
