# Supplementary material for: Dysregulated Bone Metabolism Is Related to High Expression of miR-151a-3p in Severe Adolescent Idiopathic Scoliosis
Source: Biomed Res Int. 2020 Sep 26;2020:4243015. doi: 10.1155/2020/4243015 (PMC7537684; doi:10.1155/2020/4243015)
Supplement: Supplementary Materials — Supplemental table S1: candidate miRNA expression profile by miRNA sequencing. Supplemental table S2: summary of the primer sequences for RT-PCR. Supplemental table S3: predicted target gene of miR-151a-30 by TargetScan7.2. Supplemental table S4: predicted target gene of miR-151a-30 by miRDB. Supplemental table S5: primer sequences for methylation-specific PCR (MSP). [file 4243015.f1.zip › Supplementary Table 2, 5.pdf]

Supplementary tables

Table S2. Primers for qPCR

Gene name	Primer	Sequence (5’-3’)
**miR-151a-3p**	Forward	GGATGCTAGACTGAAGCTCCT
	Reverse	CAGTGCGTGTCGTGGAGT
**miR-941**	Forward	GGCTGTGTGCACATGTG
	Reverse	GAACATGTCTGCGTATCTC
**miR-148b-5p**	Forward	GTTCTGTTATACACTCAGG
	Reverse	GAACATGTCTGCGTATCTC
**U6**	Forward	CTCGCTTCGGCAGCACA
	Reverse	AACGCTTCACGAATTTGCGT
**GAPDH**	Forward	TGTTCGTCATGGGTGTGAAC
	Reverse	ATGGCATGGACTGTGGTCAT
**GREM1**	Forward	TCATCAACCGCTTCTGTTACGGC
	Reverse	CAGAAGGAGCAGGACTGAAAGG
**ME1**	Forward	GGAGTTGCTCTTGGTGTTGTGG
	Reverse	GGATAAAGCCGACCCTCTTCCA
**PFN2**	Forward	TCGGCTACTGCGACGCCAAATA
	Reverse	GATCACTGAGCATTTCTTCGCGC
**YTHDF3**	Forward	GCTACTTTCAAGCATACCACCTC
	Reverse	ACAGGACATCTTCATACGGTTATTG
**NAMPT**	Forward	AGGGTTACAAGTTGCTGCCACC
	Reverse	CTCCACCAGAACCGAAGGCAAT
**β-actin**	Forward	ACTCTTCCAGCCTTCCTTCC
	Reverse	GTACTTGCGCTCAGGAGGAG

Table S5. Primers for MSP

Primer	Sequence	Product size (bp)
miR-151a-M-F	GAGCGTTAGGATTTATCGGG	175
miR-151a-M-R	TCGAATAAACCTATTTAACGAAACG	
miR-151a-U-F	GAGTGTTAGGATTTATTGGGG	175
miR-151a-U-R	TCAAATAAACCTATTTAACAAAACAACC	
