# Supplementary material for: Dysregulated Bone Metabolism Is Related to High Expression of miR-151a-3p in Severe Adolescent Idiopathic Scoliosis
Source: Biomed Res Int. 2020 Sep 26;2020:4243015. doi: 10.1155/2020/4243015 (PMC7537684; doi:10.1155/2020/4243015)
Supplement: Supplementary Materials — Supplemental table S1: candidate miRNA expression profile by miRNA sequencing. Supplemental table S2: summary of the primer sequences for RT-PCR. Supplemental table S3: predicted target gene of miR-151a-30 by TargetScan7.2. Supplemental table S4: predicted target gene of miR-151a-30 by miRDB. Supplemental table S5: primer sequences for methylation-specific PCR (MSP). [file 4243015.f1.zip › Supplementary table 3_TargetScan7.2.pdf]

Target gen	Representa	Gene name	3P-seq tag	Conserved	Conserved	Conserved	Conserved	Poorly con
MEX3C	ENST0000C	mex-3 RNA	5	1	1	0	0	2
SERINC4	ENST0000C	serine inco	9	0	0	0	0	2
GREM1	ENST0000C	gremlin 1, I	752	0	0	0	0	2
MS4A12	ENST0000C	membrane	10	0	0	0	0	1
PANK2	ENST0000C	pantothen	1244	1	1	0	0	0
IRF7	ENST0000C	interferon	10	0	0	0	0	1
CLK1	ENST0000C	CDC-like ki	3430	1	1	0	0	0
AC119673.	ENST00000598338.1		5	0	0	0	0	1
CYP7B1	ENST0000C	cytochrom	53	1	1	0	0	3
FBXL3	ENST0000C	F-box and l	842	0	0	0	0	2
STXBP4	ENST0000C	syntaxin bi	62	0	0	0	0	3
ME1	ENST0000C	malic enzy	586	0	0	0	0	1
KRTAP2-2	ENST0000C	keratin ass	5	0	0	0	0	1
ZNF345	ENST0000C	zinc finger	24	0	0	0	0	2
TRA2B	ENST0000C	transforme	1868	1	1	0	0	0
CEP95	ENST0000C	centrosom	46	0	0	0	0	1
ZMAT1	ENST0000C	zinc finger,	5	1	0	1	0	1
NUDT4	ENST0000C	nudix (nucl	41	0	0	0	0	2
SOCS5	ENST0000C	suppressor	72	1	1	0	0	1
UCHL1	ENST0000C	ubiquitin c	21879	0	0	0	0	1
UPP2	ENST0000C	uridine pho	5	1	1	0	0	0
ATF1	ENST0000C	activating t	1599	0	0	0	0	1
GABRA6	ENST0000C	gamma-am	5	0	0	0	0	1
RAD54B	ENST0000C	RAD54 hon	36	0	0	0	0	2
NKAIN3	ENST0000C	Na+/K+ tra	5	0	0	0	0	1
CEACAM5	ENST0000C	carcinoem	5	0	0	0	0	1
SLC26A2	ENST0000C	solute carri	748	0	0	0	0	4
PCP4L1	ENST0000C	Purkinje ce	23	0	0	0	0	3
CASP5	ENST0000C	caspase 5,	5	0	0	0	0	1
NEUROD4	ENST0000C	neuronal d	5	0	0	0	0	2
PAPOLG	ENST0000C	poly(A) pol	49	1	0	0	1	2
RPE	ENST0000C	ribulose-5-	333	0	0	0	0	2
HTR1F	ENST0000C	5-hydroxyt	5	0	0	0	0	1
SIX1	ENST0000C	SIX homeol	1264	1	1	0	0	0
PTPN12	ENST0000C	protein tyr	167	0	0	0	0	1
FAM120AC	ENST0000C	family with	258	0	0	0	0	1
AP005482.	ENST0000C	Uncharacte	44	0	0	0	0	2
RAB22A	ENST0000C	RAB22A, m	484	0	0	0	0	3
C9orf38	ENST0000C	chromoson	5	0	0	0	0	1
C1orf95	ENST0000C	chromoson	28	0	0	0	0	1
NIPAL2	ENST0000C	NIPA-like d	7	1	1	0	0	1
PFN2	ENST0000C	profilin 2	2184	1	1	0	0	0
KCNH8	ENST0000C	potassium	5	0	0	0	0	2
COL25A1	ENST0000C	collagen, ty	270	0	0	0	0	2
AQP4	ENST0000C	aquaporin	10	0	0	0	0	1
FAM169A	ENST0000C	family with	90	0	0	0	0	2

ZIK1	ENST0000C	zinc finger	64	0	0	0	0	1
LIG4	ENST0000C	ligase IV, D	113	0	0	0	0	1
ZNF24	ENST0000C	zinc finger	1796	0	0	0	0	2
WFDC11	ENST0000C	WAP four-c	5	0	0	0	0	1
AVPI1	ENST0000C	arginine va	4404	0	0	0	0	1
PGAM1	ENST0000C	phosphogly	927	0	0	0	0	1
GFM2	ENST0000C	G elongatic	19	0	0	0	0	1
RP11-382J1	ENST0000C	Uncharacte	5	0	0	0	0	1
BTLA	ENST0000C	B and T lym	5	0	0	0	0	1
RFESD	ENST0000C	Rieske (Fe-	15	1	0	1	0	2
HMGN2	ENST0000C	high mobili	184	0	0	0	0	1
KDELC2	ENST0000C	KDEL (Lys-A	667	0	0	0	0	2
LRRC37A3	ENST0000C	leucine rich	5	0	0	0	0	1
MAP3K19	ENST0000C	mitogen-ac	5	0	0	0	0	1
KLHL4	ENST0000C	kelch-like fa	58	0	0	0	0	2
DCTN4	ENST0000C	dynactin 4	153	0	0	0	0	1
SMCO1	ENST0000C	single-pass	5	0	0	0	0	1
LUC7L	ENST0000C	LUC7-like (S	898	0	0	0	0	1
YTHDF3	ENST0000C	YTH domai	444	1	1	0	0	1
AFF4	ENST0000C	AF4/FMR2	2755	0	0	0	0	3
KIAA1217	ENST0000C	KIAA1217	320	0	0	0	0	3
WNT6	ENST0000C	wingless-ty	7	0	0	0	0	1
PPP2CB	ENST0000C	protein pho	11	0	0	0	0	1
HSDL1	ENST0000C	hydroxyste	699	0	0	0	0	1
HES7	ENST0000C	hairy and e	416	0	0	0	0	1
CCDC67	ENST0000C	coiled-coil	5	1	1	0	0	0
RYBP	ENST0000C	RING1 and	342	1	0	1	0	2
CAPZA2	ENST0000C	capping pro	2065	0	0	0	0	1
SLA	ENST0000C	Src-like-ada	5	0	0	0	0	1
PGAM4	ENST0000C	phosphogly	5	0	0	0	0	1
ADAM7	ENST0000C	ADAM met	5	0	0	0	0	1
RERG	ENST0000C	RAS-like, es	162	0	0	0	0	2
KRT38	ENST0000C	keratin 38	5	0	0	0	0	1
CRK	ENST0000C	v-crk avian	1841	1	0	1	0	1
ADIPOQ	ENST0000C	adiponectir	5	0	0	0	0	1
ZNF772	ENST0000C	zinc finger	22	0	0	0	0	1
URM1	ENST0000C	ubiquitin re	345	0	0	0	0	2
KRTAP20-4	ENST0000C	keratin ass	5	0	0	0	0	1
MTPN	ENST0000C	myotrophir	1296	0	0	0	0	2
GLIPR1L2	ENST0000C	GLI pathog	5	0	0	0	0	1
AL354808.1	ENST00000601204.1		5	0	0	0	0	1
MLANA	ENST0000C	melan-A	5	0	0	0	0	1
A2ML1	ENST0000C	alpha-2-ma	5	0	0	0	0	1
HPDL	ENST0000C	4-hydroxyp	418	0	0	0	0	1
APOL6	ENST0000C	apolipopro	5	0	0	0	0	2
IKZF3	ENST0000C	IKAROS fan	7	0	0	0	0	2
IL26	ENST0000C	interleukin	5	0	0	0	0	1

ANKRD44	ENST0000C	ankyrin rep	187	0	0	0	0	1
ZNF799	ENST0000C	zinc finger	17	0	0	0	0	3
NAMPT	ENST0000C	nicotinamic	375	0	0	0	0	1
SULT4A1	ENST0000C	sulfotransf	25	0	0	0	0	2
RBM7	ENST0000C	RNA bindin	46	0	0	0	0	1
PANX3	ENST0000C	pannexin 3	5	0	0	0	0	1
C6orf106	ENST0000C	chromoson	80	1	1	0	0	0

Poorly cons	Poorly cons	Poorly cons	6mer sites	Representa	Cumulative	Total conte	Aggregate I	Previous Ta
0	1	1	1	hsa-miR-15	-0.73	-0.73	N/A	
2	0	0	0	hsa-miR-15	-0.72	-0.72	N/A	
1	0	1	0	hsa-miR-15	-0.71	-0.72	N/A	
1	0	0	0	hsa-miR-15	-0.71	-0.71	N/A	
0	0	0	0	hsa-miR-15	-0.7	-0.7	N/A	
1	0	0	0	hsa-miR-15	-0.67	-0.67	N/A	
0	0	0	0	hsa-miR-15	-0.64	-0.64	N/A	2007
1	0	0	0	hsa-miR-15	-0.64	-0.64	N/A	
0	1	2	0	hsa-miR-15	-0.64	-0.68	N/A	
1	1	0	1	hsa-miR-15	-0.62	-0.7	N/A	
1	0	2	1	hsa-miR-15	-0.62	-0.72	N/A	
1	0	0	0	hsa-miR-15	-0.62	-0.62	N/A	
1	0	0	0	hsa-miR-15	-0.61	-0.61	N/A	
1	0	1	0	hsa-miR-15	-0.61	-0.61	N/A	
0	0	0	0	hsa-miR-15	-0.59	-0.59	N/A	
1	0	0	0	hsa-miR-15	-0.59	-0.59	N/A	
0	1	0	2	hsa-miR-15	-0.57	-0.57	N/A	
1	1	0	1	hsa-miR-15	-0.57	-0.57	N/A	
1	0	0	1	hsa-miR-15	-0.57	-0.57	N/A	
1	0	0	0	hsa-miR-15	-0.56	-0.56	N/A	
0	0	0	0	hsa-miR-15	-0.56	-0.56	N/A	2007
1	0	0	2	hsa-miR-15	-0.55	-0.64	N/A	
1	0	0	0	hsa-miR-15	-0.54	-0.54	N/A	
1	0	1	0	hsa-miR-15	-0.54	-0.59	N/A	
1	0	0	0	hsa-miR-15	-0.53	-0.53	N/A	
1	0	0	0	hsa-miR-15	-0.53	-0.53	N/A	
0	1	3	2	hsa-miR-15	-0.53	-0.53	N/A	
0	0	3	0	hsa-miR-15	-0.53	-0.53	N/A	
1	0	0	0	hsa-miR-15	-0.53	-0.53	N/A	
1	0	1	0	hsa-miR-15	-0.52	-0.52	N/A	
1	0	1	1	hsa-miR-15	-0.51	-0.59	N/A	
1	0	1	0	hsa-miR-15	-0.5	-0.55	N/A	
1	0	0	1	hsa-miR-15	-0.5	-0.5	N/A	
0	0	0	0	hsa-miR-15	-0.5	-0.5	N/A	
1	0	0	0	hsa-miR-15	-0.5	-0.5	N/A	
1	0	0	1	hsa-miR-15	-0.49	-0.49	N/A	
0	2	0	0	hsa-miR-15	-0.49	-0.59	N/A	
1	0	2	1	hsa-miR-15	-0.48	-0.86	N/A	
1	0	0	0	hsa-miR-15	-0.48	-0.48	N/A	
1	0	0	0	hsa-miR-15	-0.48	-0.48	N/A	
0	0	1	1	hsa-miR-15	-0.48	-0.48	N/A	
0	0	0	0	hsa-miR-15	-0.48	-0.5	N/A	2007
1	1	0	0	hsa-miR-15	-0.47	-0.47	N/A	
2	0	0	0	hsa-miR-15	-0.47	-0.57	N/A	
1	0	0	1	hsa-miR-15	-0.47	-0.47	N/A	
0	2	0	2	hsa-miR-15	-0.47	-0.51	N/A	

1	0	0	1	hsa-miR-15	-0.47	-0.47	N/A	
1	0	0	0	hsa-miR-15	-0.47	-0.47	N/A	
1	0	1	0	hsa-miR-15	-0.47	-0.61	N/A	
0	1	0	0	hsa-miR-15	-0.47	-0.47	N/A	
1	0	0	0	hsa-miR-15	-0.47	-0.47	N/A	
1	0	0	0	hsa-miR-15	-0.47	-0.47	N/A	
1	0	0	0	hsa-miR-15	-0.47	-0.66	N/A	
1	0	0	0	hsa-miR-15	-0.47	-0.47	N/A	
1	0	0	0	hsa-miR-15	-0.47	-0.47	N/A	
0	1	1	0	hsa-miR-15	-0.46	-0.82	N/A	
1	0	0	0	hsa-miR-15	-0.46	-0.49	N/A	
1	0	1	0	hsa-miR-15	-0.46	-0.48	N/A	
1	0	0	0	hsa-miR-15	-0.46	-0.46	N/A	
1	0	0	0	hsa-miR-15	-0.46	-0.46	N/A	
0	2	0	1	hsa-miR-15	-0.45	-0.45	N/A	
1	0	0	0	hsa-miR-15	-0.45	-0.45	N/A	
1	0	0	0	hsa-miR-15	-0.45	-0.45	N/A	
1	0	0	0	hsa-miR-15	-0.45	-0.51	N/A	
0	0	1	1	hsa-miR-15	-0.45	-0.45	N/A	2007
0	0	3	0	hsa-miR-15	-0.44	-0.46	N/A	
0	2	1	0	hsa-miR-15	-0.44	-0.47	N/A	
1	0	0	0	hsa-miR-15	-0.44	-0.44	N/A	
1	0	0	0	hsa-miR-15	-0.44	-0.44	N/A	
1	0	0	0	hsa-miR-15	-0.43	-0.43	N/A	
1	0	0	0	hsa-miR-15	-0.43	-0.46	N/A	
0	0	0	0	hsa-miR-15	-0.42	-0.42	N/A	
0	2	0	0	hsa-miR-15	-0.42	-0.58	N/A	2007
1	0	0	0	hsa-miR-15	-0.42	-0.47	N/A	
1	0	0	0	hsa-miR-15	-0.42	-0.42	N/A	
1	0	0	0	hsa-miR-15	-0.42	-0.42	N/A	
1	0	0	0	hsa-miR-15	-0.42	-0.42	N/A	
0	1	1	1	hsa-miR-15	-0.41	-0.45	N/A	
1	0	0	0	hsa-miR-15	-0.4	-0.4	N/A	
0	1	0	0	hsa-miR-15	-0.4	-0.4	N/A	2007
1	0	0	1	hsa-miR-15	-0.4	-0.4	N/A	
1	0	0	1	hsa-miR-15	-0.4	-0.54	N/A	
1	0	1	0	hsa-miR-15	-0.4	-0.6	N/A	
0	1	0	0	hsa-miR-15	-0.4	-0.4	N/A	
0	1	1	0	hsa-miR-15	-0.4	-0.4	N/A	
1	0	0	0	hsa-miR-15	-0.4	-0.4	N/A	
0	1	0	0	hsa-miR-15	-0.39	-0.39	N/A	
1	0	0	0	hsa-miR-15	-0.39	-0.39	N/A	
1	0	0	0	hsa-miR-15	-0.39	-0.39	N/A	
0	1	0	0	hsa-miR-15	-0.39	-0.39	N/A	
0	0	2	1	hsa-miR-15	-0.38	-0.38	N/A	
1	1	0	0	hsa-miR-15	-0.38	-0.46	N/A	
0	1	0	0	hsa-miR-15	-0.38	-0.38	N/A	

1	0	0	0	hsa-miR-15	-0.38	-0.38	N/A	
0	0	3	1	hsa-miR-15	-0.38	-0.54	N/A	
1	0	0	1	hsa-miR-15	-0.38	-0.44	N/A	
0	1	1	0	hsa-miR-15	-0.38	-0.4	N/A	
1	0	0	0	hsa-miR-15	-0.38	-0.38	N/A	
0	1	0	1	hsa-miR-15	-0.37	-0.37	N/A	
0	0	0	0	hsa-miR-15	-0.37	-0.37	N/A	2007

rgetScan publication(s)
