# Supplementary material for: Dysregulated Bone Metabolism Is Related to High Expression of miR-151a-3p in Severe Adolescent Idiopathic Scoliosis
Source: Biomed Res Int. 2020 Sep 26;2020:4243015. doi: 10.1155/2020/4243015 (PMC7537684; doi:10.1155/2020/4243015)
Supplement: Supplementary Materials — Supplemental table S1: candidate miRNA expression profile by miRNA sequencing. Supplemental table S2: summary of the primer sequences for RT-PCR. Supplemental table S3: predicted target gene of miR-151a-30 by TargetScan7.2. Supplemental table S4: predicted target gene of miR-151a-30 by miRDB. Supplemental table S5: primer sequences for methylation-specific PCR (MSP). [file 4243015.f1.zip › Supplementray table 4_miRDB_pre_targets_miR-151a-3p.pdf]

miRDB

There are 100 top predicted targets for hsa-miR-151a-3p in miRDB.

Target Detail	Target Rank	Target Score	miRNA Name		Gene Description
Details	1	96	hsa-miR-151a-3p	FAM120AOS	family with sequence similarity 120A opposite strand
Details	2	95	hsa-miR-151a-3p	RPS6KA5	ribosomal protein S6 kinase A5
Details	3	95	hsa-miR-151a-3p	AGO2	argonaute RISC catalytic component 2
Details	4	94	hsa-miR-151a-3p	COL25A1	collagen type XXV alpha 1 chain
Details	5	94	hsa-miR-151a-3p	ME1	malic enzyme 1
Details	6	93	hsa-miR-151a-3p	UPP2	uridine phosphorylase 2
Details	7	91	hsa-miR-151a-3p	YTHDF3	YTH N6-methyladenosine RNA binding protein 3
Details	8	91	hsa-miR-151a-3p	PITPNA	phosphatidylinositol transfer protein alpha
Details	9	90	hsa-miR-151a-3p	CASD1	CAS1 domain containing 1
Details	10	89	hsa-miR-151a-3p	CLASP2	cytoplasmic linker associated protein 2
Details	11	88	hsa-miR-151a-3p	NAMPT	nicotinamide phosphoribosyltransferase
Details	12	88	hsa-miR-151a-3p	RFX3	regulatory factor X3
Details	13	88	hsa-miR-151a-3p	CLK1	CDC like kinase 1
Details	14	86	hsa-miR-151a-3p	MARC2	mitochondrial amidoxime reducing component 2
Details	15	86	hsa-miR-151a-3p	NAA15	N(alpha)-acetyltransferase 15, NatA auxiliary subunit
Details	16	85	hsa-miR-151a-3p	DCTN4	dynactin subunit 4
Details	17	85	hsa-miR-151a-3p	MAP3K19	mitogen-activated protein kinase kinase kinase 19
Details	18	84	hsa-miR-151a-3p	FXR1	FMR1 autosomal homolog 1
Details	19	83	hsa-miR-151a-3p	RILPL2	Rab interacting lysosomal protein like 2
Details	20	83	hsa-miR-151a-3p	MANEA	mannosidase endo-alpha
Details	21	83	hsa-miR-151a-3p	AQP4	aquaporin 4
Details	22	83	hsa-miR-151a-3p	SEC63	SEC63 homolog, protein translocation regulator
Details	23	83	hsa-miR-151a-3p	GRM3	glutamate metabotropic receptor 3

Details	24	83	hsa-miR-151a-3p	NIPAL2	NIPA like domain containing 2
Details	25	83	hsa-miR-151a-3p	ZNF326	zinc finger protein 326
Details	26	83	hsa-miR-151a-3p	GFM2	G elongation factor mitochondrial 2
Details	27	82	hsa-miR-151a-3p	STXBP1	syntaxin binding protein 1
Details	28	82	hsa-miR-151a-3p	NPAS3	neuronal PAS domain protein 3
Details	29	82	hsa-miR-151a-3p	ZKSCAN4	zinc finger with KRAB and SCAN domains 4
Details	30	81	hsa-miR-151a-3p	MAGEA2	MAGE family member A2
Details	31	81	hsa-miR-151a-3p	MAGEA1	MAGE family member A1
Details	32	81	hsa-miR-151a-3p	MAGEA2B	MAGE family member A2B
Details	33	81	hsa-miR-151a-3p	LRCH1	leucine rich repeats and calponin homology domain containing 1
Details	34	81	hsa-miR-151a-3p	FAM76B	family with sequence similarity 76 member B
Details	35	80	hsa-miR-151a-3p	PRKACB	protein kinase cAMP-activated catalytic subunit beta
Details	36	80	hsa-miR-151a-3p	TIAM1	T cell lymphoma invasion and metastasis 1
Details	37	79	hsa-miR-151a-3p	ADAM7	ADAM metallopeptidase domain 7
Details	38	79	hsa-miR-151a-3p	GLS	glutaminase
Details	39	78	hsa-miR-151a-3p	MBNL2	muscleblind like splicing regulator 2
Details	40	78	hsa-miR-151a-3p	TPM1	tropomyosin 1
Details	41	78	hsa-miR-151a-3p	ATP2A2	ATPase sarcoplasmic/endoplasmic reticulum Ca2+ transporting 2
Details	42	78	hsa-miR-151a-3p	FSBP	fibrinogen silencer binding protein
Details	43	78	hsa-miR-151a-3p	SIX1	SIX homeobox 1
Details	44	78	hsa-miR-151a-3p	TPR	translocated promoter region, nuclear basket protein
Details	45	77	hsa-miR-151a-3p	MORF4L2	mortality factor 4 like 2
Details	46	77	hsa-miR-151a-3p	CP	ceruloplasmin
Details	47	77	hsa-miR-151a-3p	GAB1	GRB2 associated binding protein 1
Details	48	77	hsa-miR-151a-3p	GREM1	gremlin 1, DAN family BMP antagonist
Details	49	77	hsa-miR-151a-3p	TAF5L	TATA-box binding protein associated factor 5 like
Details	50	76	hsa-miR-151a-3p	ZFAND5	zinc finger AN1-type containing 5
Details	51	76	hsa-miR-151a-3p	MEIOC	meiosis specific with coiled-coil domain
Details	52	76	hsa-miR-151a-3p	NEUROD4	neuronal differentiation 4
Details	53	76	hsa-miR-151a-3p	SHC4	SHC adaptor protein 4

Details	54	75	hsa-miR-151a-3p	UGT8	UDP glycosyltransferase 8
Details	55	75	hsa-miR-151a-3p	GABPA	GA binding protein transcription factor subunit alpha
Details	56	75	hsa-miR-151a-3p	ARMC8	armadillo repeat containing 8
Details	57	75	hsa-miR-151a-3p	NAPG	NSF attachment protein gamma
Details	58	74	hsa-miR-151a-3p	KPNA4	karyopherin subunit alpha 4
Details	59	74	hsa-miR-151a-3p	CDK16	cyclin dependent kinase 16
Details	60	74	hsa-miR-151a-3p	USP15	ubiquitin specific peptidase 15
Details	61	73	hsa-miR-151a-3p	RAD54B	RAD54 homolog B
Details	62	73	hsa-miR-151a-3p	SIAH3	siah E3 ubiquitin protein ligase family member 3
Details	63	73	hsa-miR-151a-3p	OXR1	oxidation resistance 1
Details	64	73	hsa-miR-151a-3p	GAREM1	GRB2 associated regulator of MAPK1 subtype 1
Details	65	73	hsa-miR-151a-3p	ERLIN1	ER lipid raft associated 1
Details	66	73	hsa-miR-151a-3p	TRA2B	transformer 2 beta homolog
Details	67	72	hsa-miR-151a-3p	WAPL	WAPL cohesin release factor
Details	68	72	hsa-miR-151a-3p	AKT3	AKT serine/threonine kinase 3
Details	69	72	hsa-miR-151a-3p	USF3	upstream transcription factor family member 3
Details	70	72	hsa-miR-151a-3p	NIPBL	NIPBL, cohesin loading factor
Details	71	71	hsa-miR-151a-3p	BNC2	basonuclin 2
Details	72	71	hsa-miR-151a-3p	MS4A12	membrane spanning 4-domains A12
Details	73	70	hsa-miR-151a-3p	FAM237A	family with sequence similarity 237 member A
Details	74	70	hsa-miR-151a-3p	ACTR2	ARP2 actin related protein 2 homolog
Details	75	70	hsa-miR-151a-3p	STXBP4	syntaxin binding protein 4
Details	76	70	hsa-miR-151a-3p	PFN2	profilin 2
Details	77	70	hsa-miR-151a-3p	LPIN2	lipin 2
Details	78	69	hsa-miR-151a-3p	TOPORS	TOP1 binding arginine/serine rich protein
Details	79	69	hsa-miR-151a-3p	HIPK3	homeodomain interacting protein kinase 3
Details	80	69	hsa-miR-151a-3p	GABRA6	gamma-aminobutyric acid type A receptor alpha6 subunit
Details	81	69	hsa-miR-151a-3p	DLG5	discs large MAGUK scaffold protein 5
Details	82	68	hsa-miR-151a-3p	PHACTR4	phosphatase and actin regulator 4
Details	83	68	hsa-miR-151a-3p	MAGEA4	MAGE family member A4
Details	84	68	hsa-miR-151a-3p	ZNF317	zinc finger protein 317
Details	85	68	hsa-miR-151a-3p	DCLRE1C	DNA cross-link repair 1C
Details	86	68	hsa-miR-151a-3p	FAM104A	family with sequence similarity 104 member A

Details	87	68	hsa-miR-151a-3p	CNTN2	contactin 2
Details	88	67	hsa-miR-151a-3p	HIF1A	hypoxia inducible factor 1 subunit alpha
Details	89	67	hsa-miR-151a-3p	ADAM10	ADAM metallopeptidase domain 10
Details	90	67	hsa-miR-151a-3p	RAB22A	RAB22A, member RAS oncogene family
Details	91	67	hsa-miR-151a-3p	SAP30L	SAP30 like
Details	92	67	hsa-miR-151a-3p	CHL1	cell adhesion molecule L1 like
Details	93	67	hsa-miR-151a-3p	STRN3	striatin 3
Details	94	67	hsa-miR-151a-3p	CRK	CRK proto-oncogene, adaptor protein
Details	95	67	hsa-miR-151a-3p	PTPN12	protein tyrosine phosphatase, non-receptor type 12
Details	96	66	hsa-miR-151a-3p	THBS1	thrombospondin 1
Details	97	66	hsa-miR-151a-3p	ENTPD7	ectonucleoside triphosphate diphosphohydrolase 7
Details	98	66	hsa-miR-151a-3p	NAPEPLD	N-acyl phosphatidylethanolamine phospholipase D
Details	99	65	hsa-miR-151a-3p	UBN2	ubinuclein 2
Details	100	65	hsa-miR-151a-3p	GAS7	growth arrest specific 7
